# Supplementary material for: Emerging responses implemented to prevent and respond to violence against women and children in WHO European member states during the COVID-19 pandemic: a scoping review of online media reports
Source: BMJ Open. 2021 Apr 7;11(4):e045872. doi: 10.1136/bmjopen-2020-045872 (PMC8029039; doi:10.1136/bmjopen-2020-045872)
Supplement: Supplementary data [file bmjopen-2020-045872supp002.pdf]

Supplementary Methods 1

For all searches, journals were searched for items published between 1 January 2020 and 17 September 2020.

1A: ProQuest Search Strategy

Coronavirus and abuse: (Covid OR Covid19 OR Coronavirus OR corona OR SARS-CoV-2 OR Covid 19) AND ("domestic violence" OR "domestic abuse" OR "intimate partner violence" OR "Gender based violence" OR "sexual violence" OR "femicide" OR “child abuse” OR “child maltreatment” OR “child neglect” or “child exploitation” OR “bullying” OR “trafficking” OR “sexual exploitation” OR “sexual abuse” OR “child marriage” or “youth violence” OR “infanticide” OR “stalking”)

1B: East View Information Service Search Strategy

((ковид OR COVID-19 OR COVID 19 OR Covid 19 OR коронавирус OR корона OR SARS-CoV-2 OR пандемия OR карантин OR изоляция) AND (домашнее насилие OR семейное насилие OR бытовое насилие OR побои OR насилие над женщинами OR сексуальное насилие OR насилие над детьми OR жестокое обращение с детьми OR эксплуатация детского труда OR издевательства OR травля OR торговля людьми OR сексуальное надругательство OR сексуальная эксплуатация OR брак с несовершеннолетними OR детский брак OR молодежное насилие OR детоубийство))

The search in Yandex.ru was based on the combinations of the following two search terms for “COVID-19” and “violence”:
(COVID-19 OR коронавирус) +(насилие)

Yandex.ru Search Strategy

((ковид | COVID-19 | COVID 19 | Covid 19 | коронавирус | корона | SARS-CoV-2 | пандемия | карантин | изоляция) +(домашнее насилие | семейное насилие | бытовое насилие | побои | насилие над женщинами | сексуальное насилие | насилие над детьми | жестокое обращение с детьми | эксплуатация детского труда | издевательства | травля | торговля людьми | сексуальное надругательство | сексуальная эксплуатация | брак с несовершеннолетними | детский брак | молодежное насилие | детоубийство))

1C: Ovid Search Strategy

(Covid OR Covid19 OR Coronavirus OR corona OR SARS-CoV-2 OR Covid 19) AND ("domestic violence" OR "domestic abuse" OR "intimate partner violence" OR "Gender based violence" OR "sexual violence" OR "femicide" OR “child abuse” OR “child neglect” or “child exploitation” OR “bullying” OR “trafficking” OR “sexual exploitation” OR “sexual abuse” OR “child marriage” or “youth violence” OR “infanticide” OR “stalking”)
