# Supplementary material for: Emerging responses implemented to prevent and respond to violence against women and children in WHO European member states during the COVID-19 pandemic: a scoping review of online media reports
Source: BMJ Open. 2021 Apr 7;11(4):e045872. doi: 10.1136/bmjopen-2020-045872 (PMC8029039; doi:10.1136/bmjopen-2020-045872)
Supplement: Supplementary data [file bmjopen-2020-045872supp001.pdf]

Supplementary Table 1: Number of excluded studies and reason for exclusion from the ProQuest search output

**Exclusion Reason**	**Number Excluded**
Total found from search	2,343
Total full text search	1,610
Not related to either VAW/VAC or COVID-19	329
Related to VAW/VAC but not related to COVID-19	298
Related to COVID-19 but not related to VAC/VAW	208
Insufficient Information	242
Related to VAW/VAC and COVID-19 but did not include any public health responses	252
Not relevant countries (not WHO European Member States or territories Kosovo[1] or Greenland)	74
Duplicates	47
Individual case studies	3
No access to the full text	1
Wrong date	1
**Total excluded**	**1,455**
**Total included**	**155**

[1] All references to Kosovo in this document should be understood to be in the context of the United Nations Security Council resolution 1244 (1999)

BMJ Publishing Group Limited (BMJ) disclaims all liability and responsibility arising from any reliance placed on this supplemental material which has been supplied by the author(s)

Supplementary Table 2: Type of measures reported by each of the WHO European Member States, including territories Kosovo[1] and Greenland

	Albania	Andorra	Armenia	Austria	Azerbaijan	Belarus	Belgium	Bosnia and Herzegovina	Bulgaria	Croatia	Cyprus	Czechia	Denmark	Estonia	Finland	France	Georgia	Germany	Greece	Greenland	Hungary	Iceland	Ireland	Israel	Italy	Kazakhstan	Kosovo	Kyrgyzstan	Latvia	Lithuania	Luxembourg	Malta	Monaco	Montenegro	Netherlands	North Macedonia	Norway	Poland	Portugal	Republic of Moldova	Romania	Russian Federation	San Marino	Serbia	Slovakia	Slovenia	Spain	Sweden	Switzerland	Tajikistan	Turkey	Turkmenistan	Ukraine	United Kingdom	Uzbekistan
Innovative Response	Y/N																																																						
Media campaign/Info dissemination	Y	Y	Y	Y	Y	Y	Y	Y	N	Y	Y	Y	N	Y	Y	Y	Y	Y	Y	N	N	N	Y	Y	Y	Y	Y	Y	Y	N	Y	Y	Y	Y	Y	Y	Y	Y	Y	N	Y	N	Y	Y	Y	Y	Y	Y	Y	N	Y	N	Y	Y	Y
Helpline expansion/maintain	Y	Y	N	N	Y	N	Y	Y	Y	Y	Y	N	Y	N	Y	Y	Y	Y	Y	N	N	N	Y	Y	Y	N	Y	N	N	Y	Y	Y	Y	Y	N	Y	Y	N	Y	Y	Y	Y	Y	Y	N	Y	Y	Y	N	N	Y	N	Y	Y	Y
Shelter expansion/maintain	Y	N	Y	N	N	N	Y	Y	N	Y	Y	N	Y	Y	Y	Y	Y	Y	Y	N	N	Y	Y	Y	Y	Y	Y	Y	Y	N	N	Y	N	N	Y	N	Y	Y	Y	Y	N	Y	N	Y	Y	Y	Y	N	Y	N	Y	N	Y	N	Y
Move resources online	Y	Y	N	Y	Y	N	Y	Y	N	Y	Y	N	Y	Y	N	Y	N	Y	Y	N	Y	N	Y	N	Y	N	Y	Y	Y	N	Y	Y	Y	Y	N	Y	N	Y	Y	Y	N	Y	N	Y	N	N	Y	N	N	N	Y	N	Y	Y	Y
Government guidance/policy	Y	N	N	Y	N	N	N	N	N	N	Y	N	N	N	N	Y	Y	Y	N	N	N	N	Y	Y	Y	Y	N	Y	N	Y	N	N	N	Y	N	N	N	Y	Y	Y	Y	Y	N	N	Y	N	Y	N	N	N	Y	N	Y	Y	N
App	N	N	N	N	N	N	N	N	N	N	Y	Y	N	N	N	N	N	N	N	N	N	N	N	N	Y	N	N	N	N	N	N	Y	N	Y	N	N	N	N	N	N	N	Y	Y	N	N	N	Y	N	N	N	Y	N	Y	Y	Y
Financial/goods support	Y	N	Y	N	Y	N	N	Y	N	N	N	N	N	Y	N	N	N	N	N	N	N	N	Y	N	Y	N	N	N	N	N	N	Y	N	Y	N	Y	N	N	N	N	N	N	N	N	N	N	Y	N	N	N	N	N	N	N	N
Pharmacy help point	N	N	N	N	N	N	Y	N	N	N	N	N	N	N	N	Y	N	Y	Y	N	N	N	Y	N	Y	N	Y	N	N	N	N	N	Y	N	N	N	Y	N	N	N	N	N	N	Y	N	Y	Y	N	N	N	N	N	N	N	N
Extra funding for NGOs	N	N	N	N	N	N	Y	Y	N	N	N	N	N	N	N	Y	N	N	N	N	N	Y	Y	N	Y	N	Y	N	N	N	N	N	N	N	N	N	N	N	N	N	N	N	N	Y	N	N	N	Y	N	N	N	N	N	Y	N
Advocacy	N	N	N	N	N	N	N	N	Y	N	Y	N	N	N	N	N	N	N	N	N	N	N	Y	Y	N	Y	N	Y	Y	N	N	Y	N	N	N	N	N	N	N	N	N	N	N	N	N	N	N	N	N	N	N	N	Y	N	Y
Loosen lockdown measures	Y	N	N	Y	N	N	N	N	N	N	N	N	N	N	N	N	Y	N	N	N	N	N	Y	N	Y	N	Y	N	N	N	N	Y	N	Y	Y	N	N	N	N	N	N	N	N	N	N	N	Y	N	N	N	N	N	N	Y	Y
Fast track/prioritise/extend legal processes	Y	N	N	Y	N	N	Y	N	N	Y	Y	N	N	N	N	Y	N	N	N	N	N	N	Y	N	N	N	N	Y	N	N	N	N	N	Y	N	N	N	N	N	N	N	N	N	Y	N	N	N	N	N	N	N	N	N	N	Y
Proactive contact with survivors	N	N	N	N	N	N	Y	N	N	N	N	N	N	N	N	Y	N	N	N	N	N	N	Y	N	Y	N	N	N	N	Y	N	N	N	N	N	N	N	Y	N	Y	N	N	N	Y	N	N	Y	N	N	N	N	N	N	N	N
Eviction of perpetrator	N	N	N	Y	N	N	N	N	N	N	N	Y	N	N	N	Y	N	N	N	N	N	N	N	N	Y	N	N	N	N	N	N	N	N	Y	N	N	N	N	N	N	N	N	N	N	N	N	N	N	N	N	N	N	N	N	N
Creation of taskforce	N	N	N	N	N	N	Y	N	N	N	N	N	N	N	N	N	N	N	N	N	N	N	Y	N	N	N	N	N	N	N	Y	N	N	Y	N	N	N	N	N	N	N	N	N	N	N	N	N	Y	Y	N	N	N	N	N	N
Raise police awarness	Y	Y	N	N	N	N	Y	N	N	N	N	N	N	N	Y	N	N	N	N	N	N	N	N	N	N	N	Y	N	N	N	N	N	N	N	N	N	Y	N	N	N	N	N	N	N	N	N	N	N	N	N	N	N	Y	N	N
PPE/Testing for VAWC staff	N	N	N	N	Y	N	N	N	N	N	N	N	N	N	N	N	N	N	N	N	N	N	Y	N	N	N	N	N	N	N	N	Y	N	N	N	N	N	N	N	Y	N	N	N	N	Y	N	N	N	N	N	N	N	N	Y	N
Childcare provision	N	N	N	N	N	N	N	N	N	N	N	N	N	Y	N	N	N	N	N	N	N	N	N	N	N	N	N	N	N	N	Y	N	N	N	N	N	N	N	N	N	N	N	N	N	N	Y	N	N	N	N	N	N	Y	N	N
Ensure provision of existing SRH services	N	N	N	N	N	N	N	N	N	N	N	N	N	N	N	Y	N	N	N	N	N	N	N	N	N	N	N	N	N	N	N	N	N	N	Y	N	N	N	N	N	N	N	N	N	N	N	N	N	N	N	N	N	N	Y	N
Postman checkin	N	N	N	N	N	N	N	N	N	N	N	Y	N	N	N	Y	N	N	N	N	N	N	N	N	N	N	N	N	N	N	N	N	N	N	N	N	N	N	N	N	N	N	N	N	N	N	N	N	N	N	N	N	N	Y	N
Other support for VAWC centre staff	N	N	N	N	N	N	N	N	N	N	N	N	N	N	N	N	N	N	N	N	N	N	N	N	Y	N	N	N	N	N	N	N	N	N	N	N	N	N	N	N	N	N	N	Y	N	N	N	N	N	N	N	N	N	N	N
Police prioritise DV cases	N	N	N	N	N	N	N	N	N	N	N	N	N	N	N	N	N	N	N	N	N	N	Y	N	N	N	N	N	N	N	N	N	N	N	N	N	N	N	N	N	N	N	N	N	N	N	N	N	Y	N	N	N	N	Y	N
Supermarket/shop help point	N	N	N	N	N	N	N	N	N	N	N	N	N	N	N	Y	N	N	N	N	N	N	N	N	N	N	Y	N	N	N	N	N	N	N	N	N	N	N	N	N	N	N	N	N	N	N	N	N	N	N	N	N	N	Y	N
Service coordination	Y	N	N	N	N	N	N	N	N	N	N	N	N	N	N	N	N	N	Y	N	N	N	N	N	N	Y	N	N	N	N	N	N	N	N	N	N	N	N	N	N	N	N	N	N	N	N	N	N	N	N	N	N	N	N	N
Community/corporate Fundraising	N	N	N	N	N	N	N	N	N	N	N	N	N	N	N	N	N	N	N	N	N	N	Y	N	N	N	N	N	N	N	N	N	N	N	N	N	N	N	N	N	N	Y	N	N	N	N	N	N	N	N	N	N	N	Y	N
Emergency mobile teams	N	N	N	N	N	N	N	N	N	N	N	N	N	N	N	N	N	N	N	N	N	N	N	N	N	N	N	Y	N	N	N	N	N	N	N	N	N	N	N	N	N	N	N	Y	N	N	N	N	N	N	N	N	Y	N	N
Social media codeword	N	N	N	N	N	N	N	N	N	N	N	N	N	N	N	N	N	N	N	N	N	N	N	N	Y	N	N	N	N	N	N	N	N	N	N	N	N	N	N	N	N	N	N	N	N	N	N	N	N	N	N	N	Y	N	N
Free transport	N	N	N	N	N	N	N	N	N	N	N	N	N	N	N	N	N	N	N	N	N	N	N	N	N	N	N	N	N	N	N	N	N	N	N	N	N	N	N	N	N	N	N	Y	N	N	N	N	N	N	N	N	N	Y	N
EU funding	N	N	N	N	N	N	N	N	N	N	N	N	N	N	N	N	N	N	N	N	N	N	N	N	N	N	N	Y	N	N	N	N	N	N	N	N	N	N	N	N	N	Y	N	N	N	N	N	N	N	N	N	N	N	N	N
Monitor past perpetrators	N	N	N	N	N	N	N	N	N	N	N	N	N	N	N	N	N	N	N	N	N	N	N	N	N	N	N	N	N	N	Y	N	N	N	N	N	N	N	N	N	N	N	N	N	N	Y	N	N	N	N	N	N	N	N	N
Testing for survivors	N	N	N	N	N	N	N	N	N	N	Y	N	N	N	N	N	N	N	N	N	N	N	N	N	N	N	N	N	N	N	N	N	N	N	N	N	N	N	N	N	N	N	N	N	N	N	N	N	N	N	N	N	N	N	N
Police codeword	N	N	N	N	N	N	N	N	N	N	N	N	N	N	N	N	N	N	N	N	N	N	N	N	N	Y	N	N	N	N	N	N	N	N	N	N	N	N	N	N	N	N	N	N	N	N	N	N	N	N	N	N	N	N	N
PPE for Police	N	N	N	N	N	N	N	N	N	N	N	N	N	N	N	N	N	N	N	N	N	N	N	N	N	N	N	N	N	N	N	Y	N	N	N	N	N	N	N	N	N	N	N	N	N	N	N	N	N	N	N	N	N	N	N
Open air F2F appointments	N	N	N	N	N	N	Y	N	N	N	N	N	N	N	N	N	N	N	N	N	N	N	N	N	N	N	N	N	N	N	N	N	N	N	N	N	N	N	N	N	N	N	N	N	N	N	N	N	N	N	N	N	N	N	N
Dentist guidelines for telephone assessment	N	N	N	N	N	N	N	N	N	N	N	N	N	N	N	N	N	N	N	N	N	N	N	N	N	N	N	N	N	N	N	N	N	N	N	N	N	N	N	N	N	N	N	N	N	N	N	N	N	N	N	N	N	Y	N
Medical care for refugees	N	N	N	N	N	N	N	N	N	N	N	N	N	N	N	N	N	N	N	N	N	N	N	N	N	N	N	N	N	N	N	N	N	N	N	N	N	N	Y	N	N	N	N	N	N	N	N	N	N	N	N	N	N	N	N
Alcohol ban	N	N	N	N	N	N	N	N	N	N	N	N	N	N	N	N	N	N	N	Y	N	N	N	N	N	N	N	N	N	N	N	N	N	N	N	N	N	N	N	N	N	N	N	N	N	N	N	N	N	N	N	N	N	N	N

[1] All references to Kosovo in this document should be understood to be in the context of the United Nations Security Council resolution 1244 (1999)
