# Supplementary material for: Emerging responses implemented to prevent and respond to violence against women and children in WHO European member states during the COVID-19 pandemic: a scoping review of online media reports
Source: BMJ Open. 2021 Apr 7;11(4):e045872. doi: 10.1136/bmjopen-2020-045872 (PMC8029039; doi:10.1136/bmjopen-2020-045872)
Supplement: Author's manuscript [file bmjopen-2020-045872.draft_revisions.pdf]

BMJ Open

BMJ Open is committed to open peer review. As part of this commitment we make the peer review history of every article we publish publicly available.

When an article is published we post the peer reviewers' comments and the authors' responses online. We also post the versions of the paper that were used during peer review. These are the versions that the peer review comments apply to.

The versions of the paper that follow are the versions that were submitted during the peer review process. They are not the versions of record or the final published versions. They should not be cited or distributed as the published version of this manuscript.

BMJ Open is an open access journal and the full, final, typeset and author-corrected version of record of the manuscript is available on our site with no access controls, subscription charges or pay-per-view fees (http://bmjopen.bmj.com).

If you have any questions on BMJ Open's open peer review process please email info.bmjopen@bmj.com

BMJ Open

Emerging responses implemented to prevent and respond to violence against women and children in WHO European Member States during the COVID-19 pandemic

Journal:	*BMJ Open*
Manuscript ID	bmjopen-2020-045872
Article Type:	Original research
Date Submitted by the Author:	15-Oct-2020

Complete List of Authors:	Pearson, Isabelle; London School of Hygiene & Tropical Medicine, Global Health and Development<br>Butler, Nadia; Liverpool John Moores University<br>Yelgezekova, Zhamin; World Health Organization Regional Office for Europe<br>Nihlén, Åsa ; World Health Organization Regional Office for Europe<br>Yordi Aguirre, Isabel; World Health Organization Regional Office for Europe<br>Quigg, Zara; Liverpool John Moores University<br>Stoeckl, Heidi; London School of Hygiene & Tropical Medicine, Global Health and Development

Keywords:	PUBLIC HEALTH, Child protection < PAEDIATRICS, COVID-19

SCHOLARONE™
Manuscripts

I, the Submitting Author has the right to grant and does grant on behalf of all authors of the Work (as defined in the below author licence), an exclusive licence and/or a non-exclusive licence for contributions from authors who are: i) UK Crown employees; ii) where BMJ has agreed a CC-BY licence shall apply, and/or iii) in accordance with the terms applicable for US Federal Government officers or employees acting as part of their official duties; on a worldwide, perpetual, irrevocable, royalty-free basis to BMJ Publishing Group Ltd ("BMJ") its licensees and where the relevant Journal is co-owned by BMJ to the co-owners of the Journal, to publish the Work in this journal and any other BMJ products and to exploit all rights, as set out in our licence.

The Submitting Author accepts and understands that any supply made under these terms is made by BMJ to the Submitting Author unless you are acting as an employee on behalf of your employer or a postgraduate student of an affiliated institution which is paying any applicable article publishing charge ("APC") for Open Access articles. Where the Submitting Author wishes to make the Work available on an Open Access basis (and intends to pay the relevant APC), the terms of reuse of such Open Access shall be governed by a Creative Commons licence – details of these licences and which Creative Commons licence will apply to this Work are set out in our licence referred to above.

Other than as permitted in any relevant BMJ Author's Self Archiving Policies, I confirm this Work has not been accepted for publication elsewhere, is not being considered for publication elsewhere and does not duplicate material already published. I confirm all authors consent to publication of this Work and authorise the granting of this licence.

For peer review only

Emerging responses implemented to prevent and respond to violence against women and children in WHO European Member States during the COVID-19 pandemic

Isabelle Pearson*, Gender, Violence and Health Centre, Department of Global Health and Development, London School of Hygiene and Tropical Medicine, London, UK, WC1H 9SH, Isabelle.pearson@lshtm.ac.uk

Nadia Butler, Public Health Institute, Liverpool John Moores University, Liverpool, UK

Zhamin Yelgezekova, World Health Organization Regional Office for Europe, Copenhagen, DK

Åsa Nihlen, World Health Organization Regional Office for Europe, Copenhagen, DK

Isabel Yordi Aguirre, World Health Organization Regional Office for Europe, Copenhagen, DK

Zara Quigg, Public Health Institute, Liverpool John Moores University, Liverpool, UK

Heidi Stöckl, Gender Violence & Health Centre, Department of Global Health and Development, London School of Hygiene and Tropical Medicine, London, UK.

Disclaimer: The views expressed in the article are those of the authors and do not necessarily represent the stated policy of the World Health Organization

Key words: Public Health, Gender Based Violence, COVID-19

Word Count: 5,512

* Corresponding Author

For peer review only

ABSTRACT

Objectives: This study aims to explore the strategies that governments and civil society organisations implemented to prevent and respond to the anticipated rise in VAWC during the COVID-19 pandemic.

Setting: WHO European Region

Methods: A systematic search of media reports and publications and a thorough search of other grey literature (all published 1 January to 15 June 2020) was conducted, to identify measures implemented by governments, public services and non-governmental and civil organisations to prevent or respond to VAWC during the early months of the COVID-19 pandemic.

Results: Our study found that in 52 of the 53 member states there was at least one measure, to prevent or respond to VAWC during the pandemic. Government-led or government-sponsored measures were the most common measures, reported in 46 member states. NGO and civil society-led prevention and response measures were reported in 33 member states. The most common measure was the use of media and social media to raise awareness and to provide services on VAWC through online platforms, followed by measures taken to expand and/or maintain helpline services for those exposed to violence.

Conclusion: The potential increase in VAWC during COVID-19-imposed restrictions and lockdown resulted in adaptations and/or increases in prevention and response strategies in nearly all member states. The strength of existing public health systems influenced the requirement and choice of strategies and highlights the need for sustaining and improving violence prevention and response services. Innovative strategies employed in several member states may offer opportunities for countries to strengthen prevention and responses in the near future and during similar emergencies.

For peer review only

Article Summary

Strengths and Limitations of this study

- This is the first study to use systematic methodology to conduct a media search on measures taken to prevent and respond to VAWC during COVID-19.
- This study was able to extensively search media in the Russian language and therefore provide broad coverage of many countries in Central Asia and Eastern Europe.
- Use of only English and Russian search terms means that media reports from countries where these languages are more broadly spoken will be over-represented in the results.
- Focusing largely on media reports means that innovative and unprecedented measures will be more frequently reported, meaning countries which had strong baseline measures in place already may be under-represented.

Funding Statement

This work was supported by WHO Europe grant number WHO Ref 2020/1021489

Competing Interests

The authors declare no competing interests for the study

Data Sharing Statement

All data relevant to the study are included in the article or uploaded as supplementary information

Author Contributor Statement

All authors contributed to the development of the study protocol and provided feedback on the final article. IP, NB and ZY all conducted the media, publication and grey literature searches (IP and NB in English, ZY in Russian). IP was the lead author of the article.

For peer review only

INTRODUCTION

Violence against women and children (VAWC) are important public health, gender equality and human rights issues.[1] Based on estimates by the World Health Organization (WHO), around 25% of ever partnered women in Europe have experienced sexual and/or physical violence by a partner[2] and 12% of children aged 2-17 years were reported to have experienced violence in the past 12 months or 15.2 million children.[3 4] During a pandemic, VAWC is likely to increased due to stress related to economic insecurity, quarantines and social isolation, disruptions in health and social services and increased house and care work whilst also working from home.[5 6] The outbreak of the 2019 novel coronavirus (COVID-19) has been no exception. As more and more countries went into lockdown or imposed other physical distancing restrictions, there were increasing anecdotal reports from countries of a rise in VAWC.[6 7]

Alongside other UN agencies, WHO has issued clinical and policy guidelines on responding to VAW and VAC, including during COVID-19.[8] For governments and policy makers they recommend that violence prevention and response is considered in all response plans and mitigation methods; information dissemination to the public regarding available services and increased risk factors, as well as to alert essential service providers within the community to make them aware of signs that indicate violence. They recommend governments to enforce rules and regulations around key risk factors for violence such as alcohol, drugs and weapons and to work across sectors and with civil society to coordinate support, including referral services. To support survivors, WHO recommends governments to make provisions to allow those seeking help to safely leave the home, ensure and expand helpline functions and identify ways of making services accessible remotely. The WHO guidance also includes advice to health systems managers and health providers on how to focus on VAWC in the organisation and delivery of health services, in data collection, and through preventive health measures. Recommendations include to provide information about services available locally to continue to offer first line support and medical treatment for survivors of violence through the first points of contact in health facilities in line with WHO recommendations.[8]

With these WHO recommendations on preventing and responding to VAWC during COVID-19 as a basis, the aim of this study was to investigate what measures were taken by governments, NGOs and civil society organisations across WHO European member states in the context of the COVID-19 pandemic, during the first six months of 2020.

METHODS

This study is based on a systematic search of media reports, journal articles and official organisational reports, including information from webinars and other expert meetings. Searches were conducted in English and Russian. For any article, study or report to be included, they had to include data on at least one measure implemented in the context of the COVID-19 pandemic either in response to or to prevent VAW and/or VAC. Reports also had to have been published between 1 January 2020 and 15 July 2020 and provide data for any member states of the WHO European Region, including the self-

For peer review only

governing territories of Kosovo[1] and Greenland, or data that encompassed multiple countries including at least one of the aforementioned countries. The search excluded studies that did not provide at least one measure or only recommended measures without stating that they had been implemented.

Media review

A search of online news reports in English was conducted using the International Newsstream of ProQuest, limited to the Asian, European and Middle Eastern Newsstreams. The search strategy included terms related to COVID-19 and VAWC and was restricted to the WHO European Member states with no language restrictions. A full search strategy can be found in the Supplementary Methods 1A. Reports were all exported into Excel and screened at the full text stage.

For the Russian language media reports, searches were conducted using East View Information Service. To increase coverage, an additional search was also conducted in Yandex.ru. A full search strategy for the Russian-language reports can be found in Supplementary Methods 1B. A hand search was conducted for Radio Liberty's Central Asian branches, as well as Sputniknews. Results of the hand searches were exported manually, the reviewer screened the abstract/title and exported those deemed relevant into the Excel sheet. Those included at the title and abstract stage were then screened in further detail for inclusion at the full text stage.

Publication review

A search was conducted to review any publications that have been published concerning the COVID-19 pandemic and VAWC. For the non-Russian language articles, this search was conducted in OVID, restricted to Ovid MEDLINE(R) and In-Process & Other Non-Indexed Citations and Daily and using the search strategy outlined in Supplementary Methods 1C, with no language restrictions. Publications from OVID were imported into Excel to be reviewed at the title/abstract stage and if relevant, screened at the full text stage. A separate search was conducted in Russian using Google Scholar with a translation of the search strategy: "violence AND COVID-19". Google Scholar linked to eLibrary.ru and CyberLeninka. Full texts that met the inclusion criteria were extracted into the Excel workbook. High-level organisational websites were searched in both English and Russian and any reports that met the inclusion criteria were also included for the full text review, along with any relevant information collected from organisational webinars and that occurred during this time.

Quality assurance

Three reviewers contributed to the screening of the media reports and publications and the data extraction. A trial screen and data extraction were conducted where each reviewer screened and extracted data in parallel for a subset of search results. The results of the trial screen were compared between reviewers and, once consistency was confirmed, each reviewer separately screened and extracted data from an allocated percentage of the database search results. 10% of the data extracted by each reviewer, excluding the Russian language search, was checked by a second reviewer to further ensure consistency.

---

[1] All references to Kosovo in this document should be understood to be in the context of the United Nations Security Council resolution 1244 (1999).

For peer review only - http://bmjopen.bmj.com/site/about/guidelines.xhtml

Data extraction and management

All included results were stored and analysed within an Excel workbook. For each measure, the description of the strategy was extracted and subsequently each strategy was labelled 'government led and/or sponsored' or 'NGO and civil society'. Where a measure was led by a government and at least one NGO or civil organisation, the measure was labelled as 'multiple' (please note: measures led by local NGO and civil organisations along with external country governments were labelled as 'NGO and civil society', UN agencies were considered as 'NGO and civil society' and EU funded measures were considered 'government led and/or sponsored'). Where the measure's leading body was unclear from the extracted text, a google search was conducted for clarification, if it remained unclear then it was labelled 'unclear'. Each unique measure contained in the text extract was also assigned a topic code. Topic codes were created through thematic analysis at the data extraction stage, as the researchers' understanding of the types of strategies used were developed; the topic codes used can be viewed in Table 1. These topic codes were used to group the data with similar responses. Where responses fell under multiple topic codes, the reference was duplicated so that it could fall under each code. The country, region, date of publication and the source of the data (where available) were also extracted.

Patient public involvement

No patient or public were involved

RESULTS

The ProQuest search for media reports yielded 1,255 reports (after removing duplicates) to be included in the full text search. The Russian-language media search yielded 403 results out of which 70 were considered for full text screening. Of the 1,325 screened reports, 302 media reports were included in the analysis (exclusion reasons are displayed in Supplementary Table 1). The publication search using the OVID database yielded a total of 67 publications, out of which five were included in the full text search. The Russian language database search resulted in seven publications, of which one was included. The hand searching of both Russian and non-Russian language grey literature reports of international organisations and NGOs and civil society groups resulted in 106 additional unique reports to be included in the study. The majority of measures reported in this study are from media reports and were not fact checked by the research team.

Responses implemented to protect women and children from violence during the COVID-19 pandemic

Our study found reports that in 52 out of 53 WHO European member states, at least one measure to prevent or respond to VAWC had been implemented in the context of the pandemic. The most common types of measures were government-led or government-sponsored measures, of which at least one example was reported for 46 of the 53 member states and in territories Kosovo[1] and Greenland. NGO and civil society-led responses were reported at least once in 33 of the 53 member

[1] All references to Kosovo in this document should be understood to be in the context of the United Nations Security Council resolution 1244 (1999).

For peer review only - http://bmjopen.bmj.com/site/about/guidelines.xhtml

states and in the territory if Kosovo[1]. Table 1 displays the full break down of the types of measures across countries, with further details reported in Supplementary Table 2.

Most media reports used the term domestic violence, often without clarity as to whether the term was used to encompass both children and adults. Unless stated otherwise, the term domestic violence therefore covers both women and children. In the 52 member states and two additional territories included in the results, all reported measures pertaining to 'domestic violence', 15 (28%) implemented measures on VAWC explicitly, 31 (57%) on VAW explicitly and 30 (56%) on VAC explicitly.

Government led or sponsored measures

The most frequently reported national and regional government measures were media and information dissemination campaigns, reported in 35 member states, through television, radio and social media campaigns. The Irish Department of Justice Equality, for example, collaborated with frontline services to develop TV, radio and social media advertisements highlighting their continued support for those subjected to abuse.[9] In Slovenia, the City of Ljubljana distributed leaflets with VAWC NGO contact numbers, along with the broadcasting of violence hotline numbers on public LCD screens.[10]

Regarding high-level intersectoral approaches, governments at the national and regional level created government task forces to help prevent and respond to VAWC during COVID-19. In Belgium, the Brussels and Walloon governments have created a task force in order to monitor shelters, assist actors in the field, rapidly identify emerging needs and spread information and awareness.[11] The government of Luxembourg created a crisis management system to mitigate the risk of increased VAWC.[12]

A number of countries have dedicated specific funding for measures to prevent and respond to VAWC, including the United Kingdom, France, Israel, the Republic of Ireland, Italy, Sweden, and Kosovo[1].[11 13-18] The government of Iceland also included investment in efforts to combat domestic violence in their national financial aid package announcement.[19]

Various government guidance packages and policies have been announced, either specifically for VAWC or with measures to respond to VAWC included, across member states (see Supplementary table 1 for details). This included the creation of a committee to examine the incidence of women who have being killed during lockdown in Israel; or an interagency communication strategy on VAWC during the COVID-19 crisis in Georgia.[11 20]

In terms of service coordination, multiple examples were found of government-led measures for VAWC, primarily supporting the maintenance and expansion of VAWC helplines and shelters. Methods varied from the introduction of new helplines numbers, such as text messaging numbers introduced in France and Israel, to the introduction of WhatsApp services in Spain.[11 14 21] The maintenance of shelters was also reported as a key priority for governments during this time, with measures taken to ensure they remained open or were expanded, for example, by providing them with hotels or additional accommodation.[22-28] A few governments also provided PPE and COVID-19 testing for staff and survivors and declared shelter staff as essential workers to exempt them from lockdown measures.[11 29-32]

[1] All references to Kosovo in this document should be understood to be in the context of the United Nations Security Council resolution 1244 (1999).

For peer review only

In terms of moving VAWC resources such as psychosocial support and counselling online, 11 governments announced technical-based solutions, including a new government-led email address accessible to survivors and professionals in Portugal and the French online resource, stopblues.fr, that provides support and aids reporting.[11 33] Additionally, services were set up in France to allow children facing situations of violence to directly contact authorities via SMS.[34] In Ukraine, a chatbot was set up via Telegram to provide answers to common GBV-related questions and facilitate communication with state legal aid workers, a measure reportedly popular amongst teenagers.[35]

Pharmacies and supermarkets were also used to spread information; some governments sought to physically disseminate information such as VAWC helpline numbers and relevant service providers while other governments went further and implemented pop-up counselling centres in some supermarkets.[11 24 36] Pharmacies, specifically, were also encouraged by governments to participate in the European scheme of the 'Mask-19' code word, whereby pharmacy visitors could mention 'Mask-19' if they required help for domestic violence.[11 37-40]

Several member states reported working with the police to address the expected rise in VAWC during COVID-19. In the Republic of Ireland, the police service proactively contacted every previous survivor of domestic abuse known to them[41], the government of Andorra created a video tutorial updating police officers on the VAWC guidelines protocol[11] and the Norwegian police implemented a comprehensive set of measures to react to changes in the levels of violence and ensure that police would focus on VAWC cases.[11] To ensure survivors could access required services, apps were identified for them to contact the police. In Czechia, the government disseminated the 'Bright Sky' app to allow survivors to contact support organisations and the police, and also to access advice and store evidence.[11]

Other measures focused on improving legal processes or provisions include the fast-tracking, prioritizing and extension of the legal process surrounding cases of VAWC. In particular, in Croatia allowances were made for court deadlines missed by survivors due to COVID-19, while in Serbia, the High Court Council declared that despite the courts closing, domestic violence cases would continue to be processed.[11 42] In the Russian Federation, newspapers reported the introduction of mandatory reporting, obliging the police to investigate cases of VAWC even without an official request from the victim.[43] In several other countries, governments introduced rules to ensure that in situations of abuse the perpetrator is evicted instead of the survivor[11 44-46] and in Kyrgyzstan, a bill was passed increasing detention of perpetrators of domestic violence to 48 hours, previously three hours.[47] Policies around releasing prisoners early or granting pardons under the pandemic situation excluded prisoners convicted on VAWC charges from release in a several members states.[48-53]

Some government measures were targeted at improving the response to those experiencing violence. In many countries facing stricter lockdown rules, such as Italy, Spain and Kosovo[1], governments announced that the stay home orders and strict curfews did not apply to survivors who were seeking support.[44 45 54] Furthermore, in Malta and the Republic of Ireland, women and children known to be trapped in abusive homes were reportedly provided rent supplements.[55 56] Governments in Czechia, France and the United Kingdom also promoted the use of couriers and postmen to check in with survivors of abuse.[11 18 27]

---

[1] All references to Kosovo in this document should be understood to be in the context of the United Nations Security Council resolution 1244 (1999).

For peer review only - http://bmjopen.bmj.com/site/about/guidelines.xhtml

In some cases, governments and NGOs jointly led measures to prevent and respond to VAWC. A technical group was formed in Montenegro within the European Union and UN Women-led regional programme to prepare guidance for institutions on how to deal with VAWC during COVID-19.[11] In Albania, the Women Forum Elbasan collaborated with state police to allow beneficiaries to be accompanied by police patrol, ensuring that they could access VAWC services despite the lack of transport available during the pandemic.[57] Furthermore, in Uzbekistan, a joint project with UNDP and the Ministry for Supporting Mahallas and Families prepared flyers that were distributed to pharmacies in Tashkent City and Tashkent region of Uzbekistan in order to reach vulnerable groups of the population and provide them with referral numbers in cases of violence.[58] Measures that were not attributable to an organising body but picked up in the search included the printing of VAWC helpline numbers on milk bottles in Germany.[59] In Sweden, social media app 'Snapchat' raised awareness and reached out to check in on those aged 13 to 21 who were potentially at risk of abuse and a popular Swedish landlord company distributed flyers with VAWC-related information to all its tenants.[59]

NGO and/or Civil Society led measures

Media campaigns and information dissemination were also the most frequently reported measure used by NGOs and Civil organisations to prevent and respond to VAWC during COVID-19, reported across 23 Member States plus Kosovo[1]. Their strategies varied but were most often based around radio, television and social media. For example, the Union of Women Association of Heraklion in Greece, ensured a constant presence on TV shows, news channels and radio commercials to spread awareness of VAW during COVID-19.[40] Through social media, an online awareness campaign, Stopfisha, was launched in France as a response to the suspected rise in revenge porn as a result of lockdown to help find survivors of abuse and help them to report it.[60 61] As well as raising awareness and providing service contact details, social media was also used by NGOs and civil organisations to help those in need of assistance regarding VAWC to seek help. For example, in Poland, a fictitious online cosmetic store was set up through Facebook where survivors of domestic violence could request help by pretending to order goods.[62]

Similar to the use of social media, NGOs and civil organisations in 21 countries developed or used apps and online methods to facilitate access to VAWC support services and/or to provide them with psychological and legal support through online platforms such as Zoom and Skype. NGOs across member states provided services via Telegram, WhatsApp and Viber.[18 63-68] The NGO SPAVO in Cyprus purchased 35 smart watches for women facing domestic violence that had built-in safety alarms.[69]

The maintenance (13 countries) and expansion (14 countries) of helplines and helpline services was a key measure taken by NGOs and civil society groups. NGO-led shelters in the Republic of Ireland expanded capacity by collaborating with Airbnb, in France a sports stadium was used and in Italy collaborations emerged between booking.com and a former convent.[23 70 71] In the Republic of Moldova, to overcome government-imposed quarantine measures that meant shelters were unable to accept new residents, the NGO Promo-lex rented an apartment.[13] To ensure that helplines remained functional and could meet demand, measures included increasing the number of helpline volunteers, creating chat/SMS messaging options and the expansion of helpline hours were reported.[11 57 72-77] New helplines were also set up by NGO and civil

[1] All references to Kosovo in this document should be understood to be in the context of the United Nations Security Council resolution 1244 (1999).

For peer review only

organisations in several members states.[11 76 78-82] In France the new helpline, "Don't Hit", that was launched in April to provide counselling and specialist psychological assistance to perpetrators of violence.[11] Furthermore, systems were reportedly set up to allow helpline staff to work from home and PPE and/or COVID-19 testing were made available to VAWC centre staff in several countries.[11 57] The NGO 'CAM Firenze' in Italy organized fortnightly peer-support meetings to help staff with their emotional management and well-being during the pandemic.[74] In addition, one Belgian NGO set up open air face-to-face appointments, where survivors could receive support whilst on a socially-distant walk with centre staff.[40]

Similarly to the previous measures taken by government to use pharmacies and supermarkets as help points for survivors, NGOs in North Macedonia, Italy and Greece they also used them for the physical distribution of leaflets and posters with relevant VAWC service details.[40 57 83]

Other NGO and civil organisation measures conducted in a countries only include proactive contacting and monitoring of both perpetrators (Luxembourg and Slovenia) and known survivors (Serbia, Republic of Moldova, Italy and Belgium)[11 40 57 74], food and/or hygiene packages distribution to vulnerable populations, including those affected by violence, in Malta, Naples (Italy), North Macedonia and Albania and financial and/or in-kind support to vulnerable groups in Bosnia and Herzegovina, Spain and Montenegro.[11 57 84-87] Other forms of support include the provision of free rail travel by one rail company in the United Kingdom (in partnership with NGO Women's Aid). In Serbia, where public transport was abolished during the pandemic, multiple NGOs provided private transport for survivors to access their facilities.[57] Furthermore, in the United Kingdom, dentists were called on to update their guidelines for telephone assessments under lockdown to help them continue to assess the risk of VAWC in patients with facial injuries.[88]

Measures to prevent and respond to VAWC were also implemented by other organizations. In Kosovo[1], body cameras were provided by UN Women to the police's domestic violence department to ensure sufficient evidence was collected during call outs.[89] In Serbia, the United Nations Development Programme supported public prosecutors from eight prosecution districts to organise online multi-agency meetings to process cases of VAWC.[42] The All-Ukrainian Charity Foundation, UNICEF and the United Nations Foundations supported sociopsychological assistance mobile crews in the Ukraine.[90]

DISCUSSION

This study provides an initial overview of some of the measures taken by the member states of the WHO European Region to prevent and respond to VAWC during the COVID-19 pandemic. Government responses were identified for 46 out of the 53 member states of the WHO European Region; NGOs and civil organisation-led measures were identified in 33 member states in the Region. The most frequently reported measures found in the study were media campaigns and other forms of information dissemination. Ensuring women and children are aware of the resources available to them and how the situation of the pandemic may affect their access to such resources is crucial and aligns with WHO recommendations. The high

[1] All references to Kosovo in this document should be understood to be in the context of the United Nations Security Council resolution 1244 (1999).

For peer review only

frequency of these measures may be due to political factors, and the fact that they can be rapidly arranged and implemented with a relatively low financial or resource burden. A WHO baseline assessment on health system responses to violence against women in the WHO European Region published in 2019 shows a very high proportion of WHO European member states have made national policy commitments to eliminate VAW and had national or sub-national multisectoral action plans to prevent and respond to VAW. This lays a good ground for information dissemination and media campaigns on this topic. The same assessment further found that a specific budget line for financing a health system response to VAW only existed in five European countries.

The second most reported group of measures taken by governments pertain to service coordination for survivors, in particular, the maintenance and expansion of shelters and helplines and online services for women and children facing violence. This is in line with the UN recommendations that shelters should be classified as essential services during COVID-19 and that women should have access to safe ways to seek support.[91] The need to expand or strengthen VAWC services during a pandemic could be dependent on how well these services were already supported prior to the pandemic, as well as the nature of the lock-down measures. Several countries pledged additional funds to civil society organisations to respond to the increased service needs, but our findings currently do not reveal if countries also allocated investments to strengthen public health systems services provision. Some countries took measures to facilitate physical access to services, while other moved services online or through apps.

Many of the measures taken by governments and NGO/civil organisations mentioned the continuation of counselling and psychosocial support, predominantly through the move to online methods of communication, which follows the WHO recommendation of ensuring women-centred interventions for survivors.[92] However, measures ensuring that services are available and accessible online was more often the work of NGOs and civil society organizations than of governments. Besides the movement of resources online, our study found other interesting examples of where certain measures were more often reported as being NGOs and civil organisation-led than government-led. For example, the provision of financial and good packages. Such provisions included money, food and hygiene-related items and were most often provided to groups identified by the NGOs as being vulnerable. Although not well represented in the results of this study, vulnerable groups will have likely been some of the worst affected populations during the pandemic, not only regarding VAWC. The fact that support to vulnerable groups most often reported as NGOs/civil organisation-led highlights how such populations are often under-represented in government-led responses and the important role civil society has in filling these gaps. NGOs/civil society organisations also often had to mitigate the negative impact of government-led pandemic responses. One example being the provision of transport in several member states where, in the absence of government allowances for certain groups to break lockdown rules, civil society organizations had to develop alternatives to provide assistance. Furthermore, NGOs and civil organisations were also frequently reported as leading advocacy campaigns aimed at raising awareness to VAWC in the context of the pandemic and demanding action. This highlights civil society as a crucial force for focusing attention to human rights issues and holding governments accountable.

The study did not identify any school-based measures and very few parenting programmes for the prevention and response to VAC. School-based interventions and parenting programmes are known to be effective in the prevention and reduction of VAC;[92 93] however, with widespread school closures across Europe, school-based interventions were not feasible. Further, with the current additional burden of home-schooling placed upon parents during COVID-19, the possibility of

For peer review only - http://bmjopen.bmj.com/site/about/guidelines.xhtml

conducting parenting programmes during this time was low. Schools also provide a crucial pathway for the identification and response to child maltreatment and neglect and therefore, based on the WHO guidance on VAC, it is expected that reports of VAC will increase as lockdown measures begin to lessen. As schools across the member states are now re-opening, this will be a crucial time to ensure that children are offered the necessary support and services that they may not have had access to under the pandemic restrictions.

To contextualise the results, there are some limitations of this study that should be considered. Firstly, the attention of media reports is often biased towards new and innovative strategies rather than the actions taken to maintain existing responses and systems. Therefore, countries with strong existing frameworks for VAWC prevention, who did not need to rely on novel responses, are very likely to be underrepresented in this data. Secondly, the use of only English or Russian search terms will have excluded reports in other European languages. Due to this, some countries will be overrepresented in these results, particularly the United Kingdom, Russian Federation and the Republic of Ireland. This varied representation, combined with the focus on innovative responses, means that a lack of data reported for a country should not be interpreted as a country's lack of action to protect women and children from violence during COVID-19. Similarly, due to the lack of evaluation of the presented measures, the frequency in which measures are reported here is not an indication of their effectiveness. For example, Greenland was the only jurisdiction that our study found to have enforced a regional alcohol ban for the specific purpose of preventing VAC, but due to the wealth of evidence linking alcohol consumption to VAWC, it is likely that this measure would have had a positive effect on the reduction of violence.[94 95] Furthermore, the newspaper articles do not give any indication at this stage on whether the measures had the proposed positive effect, had any adverse side effects and what level of training and support is needed to implement them. For example, using postmen to check on women at risk of VAW would require substantial training in VAW, being non-judgemental, ethics and safety. Finally, the search strategy only focused on reports explicitly referring to VAWC, thereby ignoring upstream preventative measures such as parental support, education and childcare provision that are not always mentioned in the context of VAC. This may explain the under-representation of child-focused responses in our results. Similarly, focusing only on reports that referred to violence means that many service-based measures were not widely represented in our results, such as measures to ensure access to sexual and reproductive health services, continued abortion care and access to HIV care and STI prophylaxis, all key aspects of clinical care when responding to VAW.[96]

CONCLUSION

These results provide evidence that a diverse set of measures were taken by European governments and civil society organisations to maintain and expand VAWC service provision during the first months of the pandemic. While it is clear that the COVID-19 context has led to an increased focus on VAWC, and that an overwhelming majority of countries have taken some kind of action, further research is needed on the impact of these actions and what can be learned from the past couple of months in order to "build back better". For some women and children, the living situation during lockdown is, unfortunately, not too dissimilar to pre-pandemic life living with an abuser. Therefore, rather than suggesting the implementation of the outlined emergency measures alone, governments should be encouraged to reflect on the gaps in existing national VAWC response frameworks in their countries. So, while we should celebrate the ability of governments,

For peer review only

NGOs and civil society organisations to rapidly adapt under pressure, the responsibility should now be on governments to develop stronger baseline support systems ensure that the responsibility to protect women and children does not fall on NGOs and civil society as the world rebuilds from COVID-19. Further research is also needed to understand how the mental health impacts of the pandemic, along with the wider disruptions to service provision and access to work and education will affect VAWC in the long-term.

For peer review only

REFERENCES

1. García-Moreno C, Stöckl H. Protection of sexual and reproductive health rights: addressing violence against women. *International Journal of Gynecology & Obstetrics* 2009;106(2):144-47.
2. Devries KM, Mak JY, Garcia-Moreno C, et al. The global prevalence of intimate partner violence against women. *Science* 2013;340(6140):1527-28.
3. Hillis S, Mercy J, Amobi A, et al. Global prevalence of past-year violence against children: a systematic review and minimum estimates. *Pediatrics* 2016;137(3)
4. Krug EG, Mercy JA, Dahlberg LL, et al. The world report on violence and health. *The lancet* 2002;360(9339):1083-88.
5. Cluver L, Lachman JM, Sherr L, et al. Parenting in a time of COVID-19. 2020
6. Peterman A, Potts A, O'Donnell M, et al. Pandemics and violence against women and children. *Center for Global Development working paper* 2020;528
7. Mlambo-Ngcuka P. Violence against women and girls: The shadow pandemic. *UN Women* 2020
8. World Health Organization. Addressing violence against children, women and older people during the COVID-19 pandemic: Key actions: World Health Organization, 2020.
9. Hilliard M. Efforts to help trapped domestic abuse victims. *Irish Times* 2020 2020 Apr 16;9.
10. Novak P. Ljubljana – Domestic violence awareness 2020 [Available from: https://covidnews.eurocities.eu/2020/05/06/ljubljana-domestic-violence-awareness/.
11. Council of Europe. Promoting and protecting women's rights at national level 2020 [Available from: https://www.coe.int/en/web/genderequality/promoting-and-protecting-women-s-rights.
12. The Luxembourg Chronicle. Police Respond to 243 Domestic Violence Cases during COVID-19 Crisis. *The Luxembourg Chronicle* 2020.
13. Bami XD, Nedim; Stojanovic, Milica; Marusic, Sinisa Jakov; Necsutu, Madalin; Kajosevic, Samir; Vladisavljevic, Anja; Barberá, Marcel Gascón; Todorov, Svetoslav COVID-19 and Domestic Abuse: When Home is not the Safest Place. *Balkan Insight* 2020.
14. Bisserbe NL, Pietro. As Coronavirus Piles Pressure on Families, Domestic Violence Concerns Surge. *The Wall Street Journal* 2020.
15. Horne M. BRITAIN'S MOST TRUSTED NATIONAL NEWSPAPER CORONAVIRUS SUMMARY: GLOBAL CONFIRMED CASES UK CONFIRMED CASES 4.9M 323,017 248,818 35,341 GLOBAL DEATHS UK DEATHS [SCOT REGION]. *The Times* 2020 May 20;2.
16. Mahar M. Increased Domestic Violence after COVID-19 Lockdown. *Daily Times* 2020.
17. Times of Israel. Thousands rally in Tel Aviv against violence towards women. *The Times of Israel* 2020 2020 Jun 01.
18. UN Women. COVID-19 and essential services provision for survivors of violence against women and girls. EVAW COVID-19 briefs, 2020:11.
19. Askham P. COVID-19 In Iceland: Tenth Death, New Government Package Announced. *The Reykjavík Grapevine* 2020.
20. Cashman G. The day has come. *Jerusalem Post* 2020 2020 May 24;12.
21. Winer S. Welfare Ministry: 4 suicides due to domestic violence amid coronavirus crisis. *The Times of Israel* 2020 05/03/2020
22. Abels R. Als een Française om 'masker 19' vraagt, weet de apotheker dat hij de politie moet bellen. *Trouw* 2020.
23. Cook C. Marseille club opens to victims of domestic violence. *Anadolu Agency : AA* 2020 04/21/

2020 Apr 21.
24. Dombey D, Abboud L. Victims of lockdown abuse struggle to speak out: Domestic violence. Social cost Spanish and French officials fear that despite extra schemes for women many could not get help [Asia Region]. *Financial Times* 2020 05/27/

2020 May 27;2.
25. Evers F. Extra Vlaams geld tegen huishoudelijk geweld. *DeMorgen* 2020.
26. Khan SR. Are we ready for shadow pandemic? *New Age* 2020.
27. Мухамеджанов Б. НИКАКИХ ПРОБЛЕМ: ВЛАСТЬ И ДОМАШНЕЕ НАСИЛИЕ В ЭПОХУ ПАНДЕМИИ. *Republic (Slon)* 2020.
28. Расулова О. «Пандемия внутри пандемии». Как карантин в Украине влияет на домашнее насилие. *Hromadske* 2020.
29. Gilbert D. How many coronavirus tests are being done in the UK? *Telegraphcouk* 2020 2020 May 20.

For peer review only

30. Kippenberg J. Millions of Children Threatened by Violence Risk Being Forgotten Amid Coronavirus Lockdowns. *Euronews* 2020.
31. Peter M. Coronavirus: Four new cases reported on Tuesday (updated). *Cyprus Mail* 2020 2020 May 05.
32. Wilkinson I. Spain forced to tighten its lockdown [Edition 2]. *The Times* 2020 2020 Mar 31;14.
33. Mengin A, Allé MC, Rolling J, et al. Psychopathological consequences of confinement. *Encephale* 2020;46(3S):S43-S52. doi: 10.1016/j.encep.2020.04.007 [published Online First: 2020/04/22]
34. PTI. Calls to France's child-abuse hotline surge during lockdown. *AFP International Text Wire in English* 2020 2020 Apr 09.
35. UNFPA. Silent solutions available to quarantined survivors of domestic violence 2020 [Available from: https://www.unfpa.org/news/silent-solutions-available-quarantined-survivors-domestic-violence.
36. Hymas C. Safe spaces should be set up in supermarkets for domestic abuse victims, Home Affairs committee advises. *Telegraphcouk* 2020 2020 Apr 27.
37. ANI. In COVID-19 lockdown, US reports surge in domestic violence. *Times of India* 2020.
38. Reuters News Service. UK plans codeword scheme to tackle rise in domestic abuse. *Cyprus Mail* 2020 2020 May 21.
39. Thompson R. World: Trainee doctor slayed as domestic violence reports jump amid lockdown. *Asia News Monitor* 2020 04/20/2020.
40. Conference: Perpetrator Work During COVID-19. Perpetrator Work During COVID-19; 2020; Online.
41. Begley I. Lockdown for victims of domestic abuse is a ... LIVING HELL: coronavirus crisis [Eire Region]. *Daily Mail* 2020 2020 Apr 11;10.
42. UNDP Serbia. COVID-19: UNDP urges swift action to address violence against women and girls during pandemic 2020 [Available from: https://www.rs.undp.org/content/serbia/en/home/presscenter/articles/2020/undp-poziva-na-hitnu-akciju-za-reavanje-nasilja-nad-enama-i-devo.html.
43. BBC Monitoring. Russia: Domestic violence in spotlight amid Covid-19 outbreak. *BBC Monitoring Former Soviet Union* 2020.
44. Le Page I. Fears of domestic violence rise as millions confined over virus. *CTV News* 2020.
45. Graham-Harrison EG, Angela; Smith, Helena; Ford, Liz. Lockdowns around the world bring rise in domestic violence. *The Guardian* 2020.
46. Dellanna A. France: France approves new measures to fight the Covid-19 'catastrophe' as it prepares to ease lockdown. *Asia News Monitor* 2020 2020 May 11.
47. Kloop. Kyrgyzstan acts to toughen domestic violence law. *BBC Monitoring Central Asia* 2020 2020 May 03.
48. Agence France Presse. N.Ireland to release around 200 prisoners to stem COVID-19 spread. *AFP International Text Wire in English* 2020 2020 Mar 30.
49. Daragahi B. Coronavirus: Turkey to free thousands of convicted criminals but keep journalists behind bars. *Independent* 2020.
50. Financial Mirror. COVID19: Cyprus takes action to decongest prisons. *Financial Mirror* 2020 2020 Mar 31.
51. Gradaigh PO. French warships to back up far-flung territories in virus fight. *DPA International (English)* 2020 2020 Mar 25.
52. Xinhua. Portugal to release up to 2,000 prisoners over COVID-19 pandemic. *Xinhua News Agency - CEIS* 2020 2020 Apr 10.
53. Zonshine I. Israel to release hundreds of prisoners to curb spread of coronavirus. *The Jerusalem Post (Online)* 2020 [published Online First: English ed.]
54. UN Women. Temporary shelters set up to quarantine safely domestic violence survivors in Kosovo 2020 [Available from: https://eca.unwomen.org/en/news/stories/2020/5/temporary-shelters-set-up-to-quarantine-safely-domestic-violence-survivors.
55. Bonnici O. A better tomorrow. *The Malta Independent* 2020 2020 Jun 12.
56. McMahon A, Phelan C. Rent relief for abuse victims. *The Daily Mirror* 2020 2020 Jun 15;6.
57. UN Women. Impact of the COVID-19 pandemic on specialist services for victims and survivors of violence in the Western Balkans and Turkey: A proposal for addressing the needs: UN Women, 2020:156.
58. UNDP Uzbekistan. Reducing domestic violence caused by the COVID-19 lockdown in Uzbekistan, 2020.
59. Exchange with cities on the impact of the COVID-19 crisis on women. Exchange with cities on the impact of the COVID-19 crisis on women; 2020; Online.
60. Davies S. French revenge porn activists mount 'virtual raids'. *Reuters* 2020.

For peer review only

61. Rivers D. Revenge porn soars across Europe during lockdown as cases double in Britain. *Daily Star (Online)* 2020 2020 May 06.
62. Bretan J. New domestic violence law in Poland to immediately remove perpetrators from the home. *Notes from Poland* 2020.
63. Andreou E. Coronavirus: online event this month for women in danger of domestic violence. *Cyrpus Mail* 2020.
64. Percannella A. 'A double layer of isolation': domestic abuse survivors struggling to call for help. *The Independent (Daily Edition)* 2020 2020 Apr 23;27.
65. Bouvier A. New Fortnite mission: Reaching out to abused children. *AFP International Text Wire in English* 2020 2020 Jun 16.
66. EUAM Ukraine. Domestic violence and quarantine: situation becomes more grave under lockdown, 2020.
67. Lepeska D. Turkish women's rights movement fights back as COVID-19 spurs violence. *Ahval news* 2020 2020 May 28.
68. Ukrinform. МВД запустило в Telegram чат-бот для противодействия домашнему насилию. *Ukrinform* 2020.
69. Agapiou G. Coronavirus: police say domestic violence 'not up significantly' because of lockdown. *Cyprus Mail* 2020 2020 Apr 24.
70. Davies SaB, Emma. Europe braces for domestic abuse surge 'perfect storm' amid coronavirus lockdown. *Reuters* 2020 2020 Mar 26.
71. Mangan I. Airbnb offers free stays to aid families [Eire Region]. *The Daily Mirror* 2020.
72. Agapiou G. Coronavirus: Skype seminar Thursday to discuss domestic violence. *Cyprus Mail* 2020 04/29/2020
73. Armellini A. Italy: Coronavirus lockdown raises domestic violence risks for women. *DPA International (English)* 2020 03/20/2020.
74. WWP EN. Interview with CAM Firenze. Online, 2020.
75. Gaunt M. Trauma' fear for children in lockdown. *The Yorkshire Post* 2020 2020 Apr 27;5.
76. Meagher JC, Ellen. 'There's no respite': the devastating impact of abuse during lockdown. *Irish Independent* 2020 05/09/

2020 May 09;6.
77. Shtotland I. Domestic violence in the time of corona. *Jerusalem Post* 2020 04/24/2020;22.
78. Times of Israel. Crisis hotline says thousands of Israelis calling over virus, isolation worries. *The Times of Israel* 2020 2020 Mar 14.
79. OSCE. Championing Gender in Uzbekistan: Interview with John MacGregor, OSCE Project Co-ordinator in Uzbekistan, 2020.
80. Sputnik News. Пандемия домашнего насилия: как бороться с жестокостью в семье. *Sputnik UZ* 2020.
81. Захваткина А. По-соседски: как защищают права женщин в России, Таджикистане и на Украине. *Agency of Social Information* 2020.
82. Newey SR, L; Davies, G; Gartner, A; Global Health Security Team,. Government releases more home test kits for key workers after slots run out in two minutes. *The Telegraph* 2020.
83. Grierson J. Calls for funds to house domestic violence victims during Covid-19 outbreak. *The Guardian* 2020.
84. Briguglio M. A social pact for Worker's Day – Michael Briguglio. *The Malta Independent* 2020 2020 Apr 30.
85. Jones S. 'The situation is critical': coronavirus crisis agony of Spain's poor. *The Guardian (Online)* 2020
86. UN Women. Press Release: UN Women takes initiative to ensure that women and families hit hardest by the COVID-19 crisis are not left behind in North Macedonia. Skopje, 2020.
87. Tondo L. Sex traffickers left thousands of women to starve during Italy lockdown. *The Guardian* 2020.
88. Coulthard P, Hutchison I, Bell JA, et al. COVID-19, domestic violence and abuse, and urgent dental and oral and maxillofacial surgery care. *British dental journal* 2020;228(12):923-26.
89. Webinar: Covid-19 and domestic violence against women; 2020 20 July; Online.
90. BBC Monitoring. Ukraine: Donetsk Region media highlights 13-19 Jun 20. *BBC Monitoring Former Soviet Union* 2020.
91. Guterres A. 'Make The Prevention And Redress Of Violence Against Women A Key Part Of National Response Plans For COVID-19'. *United Nations* 2020.
92. Ellsberg M, Arango DJ, Morton M, et al. Prevention of violence against women and girls: what does the evidence say? *The Lancet* 2015;385(9977):1555-66.
93. World Health Organization. INSPIRE: Seven strategies for ending violence against children: World Health Organization 2016.
94. World Health Organization. Interpersonal violence and alcohol. *World Health Organisation, Geneva* 2006

For peer review only

95. Agence France Presse. Greenland bans booze in capital over virus fears. *AFP International Text Wire in English* 2020 03/29/2020.
96. World Health Organization. Responding to intimate partner violence and sexual violence against women: WHO clinical and policy guidelines: World Health Organization 2013.

For peer review only

Table 1: Reported measures in WHO European Member States, including territories Kosovo[1] and Greenland (categorized by type, number of countries and percentage of countries)

Implemented Measure	Number of countries in which measure, was reported, overall (%)	Number of countries in which measure(s) were reported as led/sponsored by gov. (national)	Number of countries in which measure(s) were reported as led/sponsored by gov. (regional)	Number of countries in which measure(s) were reported as led by NGO/Civil society	Number of countries in which measure(s) were reported as led by NGO/Civil society and gov. in partnership	Number of countries where leading body was unclear
**Response services**						
Helpline expansion/maintain	38 (69.1)	29	3	19	2	7
Shelter expansion/maintain	36 (65.5)	22	6	13	6	8
Move resources online	34 (61.8)	10	2	21	2	7
VAWC App	13 (23.6)	9	0	5	0	2
Financial/goods support	11 (20.0)	5	1	7	0	1
Extra funding for NGOs	10 (18.2)	9	1	2	0	0
Proactive contact with survivors	9 (16.4)	5	2	4	0	0
Emergency mobile teams	3 (5.5)	1	0	1	1	0
Monitor past perpetrators	2 (3.6)	0	0	2	0	0
EU funding	2 (3.6)	2	0	0	0	0
Testing for survivors	1 (1.8)	1	0	0	0	0
Open air F2F appointments	1 (1.8)	0	0	1	0	0
**Awareness and outreach**						
Media campaign/Info dissemination	45 (81.8)	32	7	23	3	6
Official guidance/policy	23 (41.8)	19	0	0	7	0
Advocacy	10 (18.2)	2	2	8	0	0
Creation of taskforce	6 (10.9)	5	1	0	1	0
Community/corporate Fundraising	3 (5.5)	0	0	3	0	0
Social media codeword	2 (3.6)	1	0	1	0	0
**Legal support for survivors**						
Exempt from lockdown measures*	12 (21.8)	10	1	1	0	2
Fast track/prioritise/extend legal processes	10 (18.2)	9	0	3	0	2
Eviction of perpetrator	5 (9.1)	3	0	0	0	3
Police prioritise DV cases	3 (5.5)	3	0	0	0	0
Alcohol ban	1 (1.8)	0	1	0	0	0
**Coordination with other services**						
Pharmacy help point	12 (21.8)	3	3	4	0	7
Raise police awareness	7 (12.7)	5	0	1	1	0
Childcare provision	4 (7.3)	1	1	1	0	1
Postman check in	3 (5.5)	2	1	0	0	0
Supermarket/shop help point	3 (5.5)	2	0	2	0	1
Ensure provision of existing SRH services	3 (5.5)	1	1	0	0	1
Free transport	2 (3.6)	0	0	2	0	0
Police codeword	1 (1.8)	1	0	0	0	0
PPE for Police	1 (1.8)	1	0	0	0	0
Dentist guidelines for telephone assessment	1 (1.8)	0	0	1	0	0
Medical care for refugees	1 (1.8)	1	0	0	0	0
Other service coordination	3 (5.5)	0	0	2	1	0
**Strengthen capacity/protection for professionals**						
PPE/Testing for VAWC staff	6 (10.9)	2	0	4	0	0
Other support for VAWC centre staff	3 (5.5)	0	0	2	0	0

** 'Exempt from lockdown measures' refers to a situation where those facing violence within the home were exempt from the strict lockdown measures imposed in their local area, for example, being exempt from curfews or being allowed to use public transport to access support services*

[1] All references to Kosovo in this document should be understood to be in the context of the United Nations Security Council resolution 1244 (1999).

For peer review only

For peer review only

Supplementary Methods 1

For all searches, journals were searched for items published between 1 January 2020 and 15 July 2020.

1A: ProQuest Search Strategy

Coronavirus and abuse: (Covid OR Covid19 OR Coronavirus OR corona OR SARS-CoV-2 OR Covid 19) AND ("domestic violence" OR "domestic abuse" OR "intimate partner violence" OR "Gender based violence" OR "sexual violence" OR "femicide" OR “child abuse” OR “child maltreatment” OR “child neglect” or “child exploitation” OR “bullying” OR “trafficking” OR “sexual exploitation” OR “sexual abuse” OR “child marriage” or “youth violence” OR “infanticide” OR “stalking”)

1B: East View Information Service Search Strategy

((ковид OR COVID-19 OR COVID 19 OR Covid 19 OR коронавирус OR корона OR SARS-CoV-2 OR пандемия OR карантин OR изоляция) AND (домашнее насилие OR семейное насилие OR бытовое насилие OR побои OR насилие над женщинами OR сексуальное насилие OR насилие над детьми OR жестокое обращение с детьми OR эксплуатация детского труда OR издевательства OR травля OR торговля людьми OR сексуальное надругательство OR сексуальная эксплуатация OR брак с несовершеннолетними OR детский брак OR молодежное насилие OR детоубийство))

The search in Yandex.ru was based on the combinations of the following two search terms for “COVID-19” and “violence”:
(COVID-19 OR коронавирус) +(насилие)

Yandex.ru Search Strategy

((ковид | COVID-19 | COVID 19 | Covid 19 | коронавирус | корона | SARS-CoV-2 | пандемия | карантин | изоляция) +(домашнее насилие | семейное насилие | бытовое насилие | побои | насилие над женщинами | сексуальное насилие | насилие над детьми | жестокое обращение с детьми | эксплуатация детского труда | издевательства | травля | торговля людьми | сексуальное надругательство | сексуальная эксплуатация | брак с несовершеннолетними | детский брак | молодежное насилие | детоубийство))

1C: Ovid Search Strategy

(Covid OR Covid19 OR Coronavirus OR corona OR SARS-CoV-2 OR Covid 19) AND ("domestic violence" OR "domestic abuse" OR "intimate partner violence" OR "Gender based violence" OR "sexual violence" OR "femicide" OR “child abuse” OR “child neglect” or “child exploitation” OR “bullying” OR “trafficking” OR “sexual exploitation” OR “sexual abuse” OR “child marriage” or “youth violence” OR “infanticide” OR “stalking”)

For peer review only

Supplementary Table 1: Number of excluded studies and reason for exclusion from the ProQuest search output

Exclusion Reason	Number Excluded
Total found from search	1,988
Total full text search	1,325
Not related to either VAW/VAC or COVID-19	258
Related to VAW/VAC but not related to COVID-19	223
Related to COVID-19 but not related to VAC/VAW	149
Insufficient Information	214
Related to VAW/VAC and COVID-19 but did not include any public health responses	83
Not relevant countries (not WHO European Member States or territories Kosovo[1] or Greenland)	59
Duplicates	42
Individual case studies	2
No access to the full text	1
Wrong date	1
**Total excluded**	**1,023**
**Total included**	**302**

[1] All references to Kosovo in this document should be understood to be in the context of the United Nations Security Council resolution 1244 (1999)

For peer review only - http://bmjopen.bmj.com/site/about/guidelines.xhtml

Supplementary Table 2: Type of measures reported by each of the WHO European Member States, including territories Kosovo[1] and Greenland

	Albania	Andorra	Armenia	Austria	Azerbaijan	Belarus	Belgium	Bosnia and Herzegovina	Bulgaria	Croatia	Cyprus	Czechia	Denmark	Estonia	Finland	France	Georgia	Germany	Greece	Greenland	Hungary	Iceland	Ireland	Israel	Italy	Kazakhstan	Kosovo	Kyrgyzstan	Latvia
Innovative Response	Y/N																												
Media campaign/Info dissemination	Y	Y	Y	Y	Y	Y	Y	Y	N	Y	Y	Y	N	Y	Y	Y	Y	Y	Y	N	N	N	Y	Y	Y	Y	Y	Y	Y
Helpline expansion/maintain	Y	Y	N	N	Y	N	Y	Y	Y	Y	Y	N	Y	N	Y	Y	Y	Y	Y	N	N	N	Y	Y	Y	N	Y	N	N
Shelter expansion/maintain	Y	N	Y	N	N	N	Y	Y	N	Y	Y	N	Y	Y	Y	Y	Y	Y	Y	N	N	Y	Y	Y	Y	Y	Y	Y	Y
Move resources online	Y	Y	N	Y	Y	N	Y	Y	N	Y	Y	N	Y	Y	N	Y	N	Y	Y	N	Y	N	Y	N	Y	N	Y	Y	Y
Government guidance/policy	Y	N	N	Y	N	N	N	N	N	N	Y	N	N	N	N	Y	Y	Y	N	N	N	N	Y	Y	Y	Y	N	Y	N
App	N	N	N	N	N	N	N	N	N	N	Y	Y	N	N	N	N	N	N	N	N	N	N	N	N	Y	N	N	N	N
Financial/goods support	Y	N	Y	N	Y	N	N	Y	N	N	N	N	N	Y	N	N	N	N	N	N	N	N	Y	N	Y	N	N	N	N
Pharmacy help point	N	N	N	N	N	N	Y	N	N	N	N	N	N	N	N	Y	N	Y	Y	N	N	N	Y	N	Y	N	Y	N	N
Extra funding for NGOs	N	N	N	N	N	N	Y	Y	N	N	N	N	N	N	N	Y	N	N	N	N	N	Y	Y	N	Y	N	Y	N	N
Advocacy	N	N	N	N	N	N	N	N	Y	N	Y	N	N	N	N	N	N	N	N	N	N	N	Y	Y	N	Y	N	Y	Y
Loosen lockdown measures	Y	N	N	Y	N	N	N	N	N	N	N	N	N	N	N	N	Y	N	N	N	N	N	Y	N	Y	N	Y	N	N
Fast track/prioritise/extend legal processes	Y	N	N	Y	N	N	Y	N	N	Y	Y	N	N	N	N	Y	N	N	N	N	N	N	Y	N	N	N	N	Y	N
Proactive contact with survivors	N	N	N	N	N	N	Y	N	N	N	N	N	N	N	N	Y	N	N	N	N	N	N	Y	N	Y	N	N	N	N
Eviction of perpetrator	N	N	N	Y	N	N	N	N	N	N	N	Y	N	N	N	Y	N	N	N	N	N	N	N	N	Y	N	N	N	N
Creation of taskforce	N	N	N	N	N	N	Y	N	N	N	N	N	N	N	N	N	N	N	N	N	N	N	Y	N	N	N	N	N	N
Raise police awarness	Y	Y	N	N	N	N	Y	N	N	N	N	N	N	N	Y	N	N	N	N	N	N	N	N	N	N	N	Y	N	N
PPE/Testing for VAWC staff	N	N	N	N	Y	N	N	N	N	N	N	N	N	N	N	N	N	N	N	N	N	N	Y	N	N	N	N	N	N
Childcare provision	N	N	N	N	N	N	N	N	N	N	N	N	N	Y	N	N	N	N	N	N	N	N	N	N	N	N	N	N	N
Ensure provision of existing SRH services	N	N	N	N	N	N	N	N	N	N	N	N	N	N	N	Y	N	N	N	N	N	N	N	N	N	N	N	N	N
Postman checkin	N	N	N	N	N	N	N	N	N	N	N	Y	N	N	N	Y	N	N	N	N	N	N	N	N	N	N	N	N	N
Other support for VAWC centre staff	N	N	N	N	N	N	N	N	N	N	N	N	N	N	N	N	N	N	N	N	N	N	N	N	Y	N	N	N	N
Police prioritise DV cases	N	N	N	N	N	N	N	N	N	N	N	N	N	N	N	N	N	N	N	N	N	N	Y	N	N	N	N	N	N
Supermarket/shop help point	N	N	N	N	N	N	N	N	N	N	N	N	N	N	N	Y	N	N	N	N	N	N	N	N	N	N	Y	N	N
Service coordination	Y	N	N	N	N	N	N	N	N	N	N	N	N	N	N	N	N	N	Y	N	N	N	N	N	N	Y	N	N	N
Community/corporate Fundraising	N	N	N	N	N	N	N	N	N	N	N	N	N	N	N	N	N	N	N	N	N	N	Y	N	N	N	N	N	N
Emergency mobile teams	N	N	N	N	N	N	N	N	N	N	N	N	N	N	N	N	N	N	N	N	N	N	N	N	N	N	N	Y	N
Social media codeword	N	N	N	N	N	N	N	N	N	N	N	N	N	N	N	N	N	N	N	N	N	N	N	N	Y	N	N	N	N
Free transport	N	N	N	N	N	N	N	N	N	N	N	N	N	N	N	N	N	N	N	N	N	N	N	N	N	N	N	N	N
EU funding	N	N	N	N	N	N	N	N	N	N	N	N	N	N	N	N	N	N	N	N	N	N	N	N	N	N	N	Y	N
Monitor past perpetrators	N	N	N	N	N	N	N	N	N	N	N	N	N	N	N	N	N	N	N	N	N	N	N	N	N	N	N	N	N
Testing for survivors	N	N	N	N	N	N	N	N	N	N	Y	N	N	N	N	N	N	N	N	N	N	N	N	N	N	N	N	N	N
Police codeword	N	N	N	N	N	N	N	N	N	N	N	N	N	N	N	N	N	N	N	N	N	N	N	N	N	Y	N	N	N
PPE for Police	N	N	N	N	N	N	N	N	N	N	N	N	N	N	N	N	N	N	N	N	N	N	N	N	N	N	N	N	N
Open air F2F appointments	N	N	N	N	N	N	Y	N	N	N	N	N	N	N	N	N	N	N	N	N	N	N	N	N	N	N	N	N	N
Dentist guidelines for telephone assessment	N	N	N	N	N	N	N	N	N	N	N	N	N	N	N	N	N	N	N	N	N	N	N	N	N	N	N	N	N
Medical care for refugees	N	N	N	N	N	N	N	N	N	N	N	N	N	N	N	N	N	N	N	N	N	N	N	N	N	N	N	N	N
Alcohol ban	N	N	N	N	N	N	N	N	N	N	N	N	N	N	N	N	N	N	N	Y	N	N	N	N	N	N	N	N	N

	Lithuania	Luxembourg	Malta	Monaco	Montenegro	Netherlands	North Macedonia	Norway	Poland	Portugal	Republic of Moldova	Romania	Russian Federation	San Marino	Serbia	Slovakia	Slovenia	Spain	Sweden	Switzerland	Tajikistan	Turkey	Turkmenistan	Ukraine	United Kingdom	Uzbekistan
Innovative Response																										
Media campaign/Info dissemination	N	Y	Y	Y	Y	Y	Y	Y	Y	Y	N	Y	N	Y	Y	Y	Y	Y	Y	Y	N	Y	N	Y	Y	Y
Helpline expansion/maintain	Y	Y	Y	Y	Y	N	Y	Y	N	Y	Y	Y	Y	Y	Y	N	Y	Y	Y	N	N	Y	N	Y	Y	Y
Shelter expansion/maintain	N	N	Y	N	N	Y	N	Y	Y	Y	Y	N	Y	N	Y	Y	Y	Y	N	Y	N	Y	N	Y	N	Y
Move resources online	N	Y	Y	Y	Y	N	Y	N	Y	Y	Y	N	Y	N	Y	N	N	Y	N	N	N	Y	N	Y	Y	Y
Government guidance/policy	Y	N	N	N	Y	N	N	N	Y	Y	Y	Y	Y	N	N	Y	N	Y	N	N	N	Y	N	Y	Y	N
App	N	N	Y	N	Y	N	N	N	N	N	N	N	Y	Y	N	N	N	Y	N	N	N	Y	N	Y	Y	Y
Financial/goods support	N	N	Y	N	Y	N	Y	N	N	N	N	N	N	N	N	N	N	Y	N	N	N	N	N	N	N	N
Pharmacy help point	N	N	N	Y	N	N	N	Y	N	N	N	N	N	N	Y	N	Y	Y	N	N	N	N	N	N	N	N
Extra funding for NGOs	N	N	N	N	N	N	N	N	N	N	N	N	N	N	Y	N	N	N	Y	N	N	N	N	N	Y	N
Advocacy	N	N	Y	N	N	N	N	N	N	N	N	N	N	N	N	N	N	N	N	N	N	N	N	Y	N	Y
Loosen lockdown measures	N	N	Y	N	Y	Y	N	N	N	N	N	N	N	N	N	N	N	Y	N	N	N	N	N	N	Y	Y
Fast track/prioritise/extend legal processes	N	N	N	N	Y	N	N	N	N	N	N	N	N	N	Y	N	N	N	N	N	N	N	N	N	N	Y
Proactive contact with survivors	Y	N	N	N	N	N	N	N	Y	N	Y	N	N	N	Y	N	N	Y	N	N	N	N	N	N	N	N
Eviction of perpetrator	N	N	N	N	Y	N	N	N	N	N	N	N	N	N	N	N	N	N	N	N	N	N	N	N	N	N
Creation of taskforce	N	Y	N	N	Y	N	N	N	N	N	N	N	N	N	N	N	N	N	Y	Y	N	N	N	N	N	N
Raise police awarness	N	N	N	N	N	N	N	Y	N	N	N	N	N	N	N	N	N	N	N	N	N	N	N	Y	N	N
PPE/Testing for VAWC staff	N	N	Y	N	N	N	N	N	N	N	Y	N	N	N	N	Y	N	N	N	N	N	N	N	N	Y	N
Childcare provision	N	Y	N	N	N	N	N	N	N	N	N	N	N	N	N	N	Y	N	N	N	N	N	N	Y	N	N
Ensure provision of existing SRH services	N	N	N	N	N	Y	N	N	N	N	N	N	N	N	N	N	N	N	N	N	N	N	N	N	Y	N
Postman checkin	N	N	N	N	N	N	N	N	N	N	N	N	N	N	N	N	N	N	N	N	N	N	N	N	Y	N
Other support for VAWC centre staff	N	N	N	N	N	N	N	N	N	N	N	N	N	N	Y	N	N	N	N	N	N	N	N	N	N	N
Police prioritise DV cases	N	N	N	N	N	N	N	N	N	N	N	N	N	N	N	N	N	N	N	Y	N	N	N	N	Y	N
Supermarket/shop help point	N	N	N	N	N	N	N	N	N	N	N	N	N	N	N	N	N	N	N	N	N	N	N	N	Y	N
Service coordination	N	N	N	N	N	N	N	N	N	N	N	N	N	N	N	N	N	N	N	N	N	N	N	N	N	N
Community/corporate Fundraising	N	N	N	N	N	N	N	N	N	N	N	N	Y	N	N	N	N	N	N	N	N	N	N	N	Y	N
Emergency mobile teams	N	N	N	N	N	N	N	N	N	N	N	N	N	N	Y	N	N	N	N	N	N	N	N	Y	N	N
Social media codeword	N	N	N	N	N	N	N	N	N	N	N	N	N	N	N	N	N	N	N	N	N	N	N	Y	N	N
Free transport	N	N	N	N	N	N	N	N	N	N	N	N	N	N	Y	N	N	N	N	N	N	N	N	N	Y	N
EU funding	N	N	N	N	N	N	N	N	N	N	N	N	Y	N	N	N	N	N	N	N	N	N	N	N	N	N
Monitor past perpetrators	N	Y	N	N	N	N	N	N	N	N	N	N	N	N	N	N	Y	N	N	N	N	N	N	N	N	N
Testing for survivors	N	N	N	N	N	N	N	N	N	N	N	N	N	N	N	N	N	N	N	N	N	N	N	N	N	N
Police codeword	N	N	N	N	N	N	N	N	N	N	N	N	N	N	N	N	N	N	N	N	N	N	N	N	N	N
PPE for Police	N	N	Y	N	N	N	N	N	N	N	N	N	N	N	N	N	N	N	N	N	N	N	N	N	N	N
Open air F2F appointments	N	N	N	N	N	N	N	N	N	N	N	N	N	N	N	N	N	N	N	N	N	N	N	N	N	N
Dentist guidelines for telephone assessment	N	N	N	N	N	N	N	N	N	N	N	N	N	N	N	N	N	N	N	N	N	N	N	N	Y	N
Medical care for refugees	N	N	N	N	N	N	N	N	N	Y	N	N	N	N	N	N	N	N	N	N	N	N	N	N	N	N
Alcohol ban	N	N	N	N	N	N	N	N	N	N	N	N	N	N	N	N	N	N	N	N	N	N	N	N	N	N

[1] All references to Kosovo in this document should be understood to be in the context of the United Nations Security Council resolution 1244 (1999)

For peer review only - http://bmjopen.bmj.com/site/about/guidelines.xhtml

BMJ Open

Emerging responses implemented to prevent and respond to violence against women and children in WHO European Member States during the COVID-19 pandemic: a scoping review of online media reports

Journal:	*BMJ Open*
Manuscript ID	bmjopen-2020-045872.R1
Article Type:	Original research
Date Submitted by the Author:	21-Nov-2020
Complete List of Authors:	Pearson, Isabelle; London School of Hygiene & Tropical Medicine, Global Health and Development<br>Butler, Nadia; Liverpool John Moores University<br>Yelgezekova, Zhamin; World Health Organization Regional Office for Europe<br>Nihlén, Åsa ; World Health Organization Regional Office for Europe<br>Yordi Aguirre, Isabel; World Health Organization Regional Office for Europe<br>Quigg, Zara; Liverpool John Moores University<br>Stoeckl, Heidi; London School of Hygiene & Tropical Medicine, Global Health and Development
**Primary Subject Heading**:	Public health
Secondary Subject Heading:	Epidemiology
Keywords:	PUBLIC HEALTH, Child protection < PAEDIATRICS, COVID-19

SCHOLARONE™
Manuscripts

I, the Submitting Author has the right to grant and does grant on behalf of all authors of the Work (as defined in the below author licence), an exclusive licence and/or a non-exclusive licence for contributions from authors who are: i) UK Crown employees; ii) where BMJ has agreed a CC-BY licence shall apply, and/or iii) in accordance with the terms applicable for US Federal Government officers or employees acting as part of their official duties; on a worldwide, perpetual, irrevocable, royalty-free basis to BMJ Publishing Group Ltd ("BMJ") its licensees and where the relevant Journal is co-owned by BMJ to the co-owners of the Journal, to publish the Work in this journal and any other BMJ products and to exploit all rights, as set out in our licence.

The Submitting Author accepts and understands that any supply made under these terms is made by BMJ to the Submitting Author unless you are acting as an employee on behalf of your employer or a postgraduate student of an affiliated institution which is paying any applicable article publishing charge ("APC") for Open Access articles. Where the Submitting Author wishes to make the Work available on an Open Access basis (and intends to pay the relevant APC), the terms of reuse of such Open Access shall be governed by a Creative Commons licence – details of these licences and which Creative Commons licence will apply to this Work are set out in our licence referred to above.

Other than as permitted in any relevant BMJ Author's Self Archiving Policies, I confirm this Work has not been accepted for publication elsewhere, is not being considered for publication elsewhere and does not duplicate material already published. I confirm all authors consent to publication of this Work and authorise the granting of this licence.

For peer review only

Emerging responses implemented to prevent and respond to violence against women and children in WHO European Member States during the COVID-19 pandemic: a scoping review of online media reports

Isabelle Pearson*, Gender, Violence and Health Centre, Department of Global Health and Development, London School of Hygiene and Tropical Medicine, London, UK, WC1H 9SH, Isabelle.pearson@lshtm.ac.uk

Nadia Butler, Public Health Institute, Liverpool John Moores University, Liverpool, UK

Zhamin Yelgezekova, World Health Organization Regional Office for Europe, Copenhagen, DK

Åsa Nihlen, World Health Organization Regional Office for Europe, Copenhagen, DK

Isabel Yordi Aguirre, World Health Organization Regional Office for Europe, Copenhagen, DK

Zara Quigg, Public Health Institute, Liverpool John Moores University, Liverpool, UK

Heidi Stöckl, Gender Violence & Health Centre, Department of Global Health and Development, London School of Hygiene and Tropical Medicine, London, UK.

Disclaimer: The views expressed in the article are those of the authors and do not necessarily represent the stated policy of the World Health Organization

Key words: Public Health, Gender Based Violence, COVID-19

Word Count: 5,549

* Corresponding Author

For peer review only

ABSTRACT

Objectives: This study aims to explore the strategies that governments and civil society organisations implemented to prevent and respond to the anticipated rise in violence against women and/or children (VAWC) during the 2019 novel coronavirus (COVID-19) pandemic.

Design: A scoping review and content analysis of online media reports.

Setting: WHO European Region.

Methods: A scoping review of media reports and publications and a search of other grey literature (published 1 January to 17 September 2020). Primary and secondary outcome measures included measures implemented by governments, public services and non-governmental and civil organisations to prevent or respond to VAWC during the early months of the COVID-19 pandemic.

Results: Our study found that in 52 of the 53 member states there was at least one measure undertaken to prevent or respond to VAWC during the pandemic. Government-led or government-sponsored measures were the most common, reported in 50 member states. Non-governmental and other civil society-led prevention and response measures were reported in 40 member states. The most common measure was the use of media and social media to raise awareness of VAWC and to provide services on VAWC through online platforms, followed by measures taken to expand and/or maintain helpline services for those exposed to violence.

Conclusion: The potential increase in VAWC during COVID-19-imposed restrictions and lockdowns resulted in adaptations and/or increases in prevention and response strategies in nearly all member states. The strength of existing public health systems influenced the requirement and choice of strategies and highlights the need for sustaining and improving violence prevention and response services. Innovative strategies employed in several member states may offer opportunities for countries to strengthen prevention and responses in the near future and during similar emergencies.

For peer review only

Article Summary

Strengths and limitations of this study

- This is the first study to use a systematic methodology to conduct a media search on measures taken to prevent and respond to VAWC during COVID-19.
- This study was able to extensively search media in the Russian language and therefore provide broad coverage of many countries in Central Asia and Eastern Europe.
- Use of only English and Russian search terms means that media reports from countries where these languages are widely spoken will be over-represented in the results.
- Focusing largely on media reports means that innovative and unprecedented measures will be more frequently reported, thus countries which had strong baseline measures in place already may be under-represented.

Funding Statement

This work was supported by WHO Europe grant number WHO Ref 2020/1021489

Competing Interests

The authors declare no competing interests for the study

Data Sharing Statement

All data relevant to the study are included in the article or uploaded as supplementary information

Author Contributor Statement

IP, NB, ZY, ZQ, AN, IYA and HS all contributed to the development of the study protocol, the planning of the paper and provided feedback on all drafts of the paper. IP, NB and ZY all conducted the media, publication and grey literature searches (IP and NB in English, ZY in Russian). IP, NB and ZY conducted the content analysis. IP, NB, ZY, ZQ, AN, IYA and HS edited the final manuscript. IP was the lead author of the article. All authors approved the final manuscript.

For peer review only

INTRODUCTION

Violence against women and children (VAWC) is an important public health, gender equality and human rights issue.[1] Based on estimates by the World Health Organization (WHO), around 25% of ever partnered women in Europe have experienced sexual and/or physical violence by a partner[2] and 12% of children aged 2-17 years were reported to have experienced violence in the past 12 months (around 15.2 million children).[3 4] During a pandemic, VAWC is likely to increase due to stress related to economic insecurity, quarantine and social isolation, disruptions in health and social services and increased house and care work whilst working from home.[5 6] The outbreak of the 2019 novel coronavirus (COVID-19) has been no exception. As more and more countries went into lockdown or imposed other physical distancing restrictions, there were increasing anecdotal reports from countries of a rise in VAWC.[6 7]

Alongside other UN agencies, WHO has issued clinical and policy guidelines on responding to violence against women (VAW) and violence against children (VAC), including during COVID-19.[8] For governments and policy makers, they recommend that violence prevention and response is considered in all response plans and mitigation methods; information dissemination to the public regarding available services and increased risk factors, as well as to alert essential service providers within the community to make them aware of signs that indicate violence. They recommend governments enforce rules and regulations around key risk factors for violence such as alcohol, drugs and weapons and to work across sectors and with civil society to coordinate support, including referral services. To support survivors, WHO recommends governments make provisions to allow those seeking help to safely leave the home, ensure and expand helpline functions and identify ways of making services accessible remotely. The WHO guidance also includes advice to health systems managers and health providers on how to focus on VAWC in the organisation and delivery of health services, in data collection and through preventive health measures. Recommendations include providing information about services available locally that continue to offer first line support and medical treatment for survivors of violence through the first points of contact in health facilities, in line with WHO recommendations.[8]

With these WHO recommendations on preventing and responding to VAWC during COVID-19 as a basis, the aim of this study was to investigate what measures were taken by governments, NGOs and other civil society organisations across WHO European member states in the context of the COVID-19 pandemic, during the first nine months of 2020.

METHODS

This study is based on a scoping review of media reports, journal articles and official organisational reports, including information from webinars and other expert meetings. Searches were conducted in English and Russian. For any article, study or report to be included, they had to include data on at least one measure implemented in the context of the COVID-19 pandemic, either in response to or to prevent VAW and/or VAC. Reports also had to have been published between 1 January 2020 and 17 September 2020 and provide data for any member state of the WHO European Region, including the

For peer review only - http://bmjopen.bmj.com/site/about/guidelines.xhtml

self-governing territories of Kosovo[1] and Greenland, or data that encompassed multiple countries including at least one of the aforementioned countries. The search excluded reports that did not provide at least one measure or only recommended measures without stating that they had been implemented.

Media review

A review of media reports published between 1 January 2020 and 17 September 2020 was conducted. A search of online news reports in English was conducted using the International Newsstream of ProQuest, limited to the Asian, European and Middle Eastern Newsstreams. The search strategy included terms related to COVID-19 and VAWC and was restricted to the WHO European member states, with no language restrictions. A full search strategy can be found in the Supplementary Methods 1A. Reports were all exported into Excel and screened at the full text stage.

For the Russian language media reports, searches were conducted using East View Information Service. To increase coverage, an additional search was conducted in Yandex.ru. A full search strategy for the Russian-language reports can be found in Supplementary Methods 1B. A hand search was conducted for Radio Liberty's Central Asian branches, as well as Sputniknews. Results of the hand searches were exported manually, the reviewer screened the abstract/title and exported those deemed relevant into the Excel sheet. Those included at the title and abstract stage were then screened in further detail for inclusion at the full text stage.

Publication review

A search was conducted to review any publications that were published between 1 January 2020 and 17 September 2020 concerning the COVID-19 pandemic and VAWC. For the non-Russian language articles, this search was conducted in OVID, restricted to Ovid MEDLINE(R) and In-Process & Other Non-Indexed Citations and Daily and using the search strategy outlined in Supplementary Methods 1C, with no language restrictions. Publications from OVID were imported into Excel to be reviewed at the title/abstract stage and if relevant, screened at the full text stage. A separate search was conducted in Russian using Google Scholar with a translation of the search strategy. Google Scholar linked to eLibrary.ru and CyberLeninka. Full texts that met the inclusion criteria were extracted into the Excel workbook. High-level organisational websites were searched in both English and Russian and any reports that met the inclusion criteria were also included for the full text review, along with any relevant information collected from organisational webinars that occurred during this time and were attended/accessible to the research team.

Quality assurance

Three reviewers contributed to the screening of the media reports and publications and data extraction. A trial screen and data extraction were conducted where each reviewer screened and extracted data in parallel for a subset of search results. The results of the trial screen were compared between reviewers and, once consistency was confirmed, each reviewer

[1] All references to Kosovo in this document should be understood to be in the context of the United Nations Security Council resolution 1244 (1999).

For peer review only - http://bmjopen.bmj.com/site/about/guidelines.xhtml

separately screened and extracted data from an allocated percentage of the database search results. 10% of the data extracted by each reviewer, excluding the Russian language search, was checked by a second reviewer to further ensure consistency.

Data extraction and management

All included results were stored and analysed within an Excel workbook. For each measure, the description of the strategy was extracted and subsequently each strategy was labelled 'government led and/or sponsored' or 'NGO and civil society'. Where a measure was led by a government and at least one NGO or civil organisation, the measure was labelled as 'multiple' (please note: measures led by local NGO and civil organisations along with external country governments were labelled as 'NGO and civil society', UN agencies were considered as 'NGO and civil society' and EU funded measures were considered 'government led and/or sponsored'). Where the measure's leading body was unclear from the extracted text, a google search was conducted for clarification, and if it remained unclear then it was labelled 'unclear'. Each unique measure contained in the text extract was also assigned a topic code. Topic codes were created through thematic analysis at the data extraction stage as the researchers' understanding of the types of strategies used were developed. These topic codes were used to group the data with similar responses. Where responses fell under multiple topic codes, the reference was duplicated so that it could fall under each code. The country, region, date of publication and the source of the data (where available) were also extracted.

Patient public involvement

No patient or public member were involved in the study.

RESULTS

The ProQuest search for media reports yielded 1,610 reports (after removing duplicates) to be included in the full text search. The Russian-language media search yielded 95 results that were considered for full text screening. Of the 1,705 screened reports, 187 media reports were included in the analysis (exclusion reasons are displayed in Supplementary Table 1). The publication search using the OVID database yielded a total of 157 publications, out of which six were included in the analysis. The Russian language database search resulted in seven publications, of which one was included. The hand searching of both Russian and non-Russian language grey literature reports of international organisations and NGOs and other civil society groups resulted in 158 additional unique reports to be included in the study. The majority of measures reported in this study are from media reports and we rely only on the information those media reports provided. The research team did not conduct further checks on the information provided. The publications found did not include any primary data collection regarding measures to prevent and respond to VAWC during COVID-19. They did, however, cite additional measures announced through media platforms, most often online newspaper articles. Due to this, the publications were used to supplement the results of our media search with the additional media reports cited through the publications, and did not offer any examples of primary data analyses.

For peer review only

Responses implemented to protect women and children from violence during the COVID-19 pandemic

Our study found reports to illustrate that in 52 out of 53 WHO European member states, at least one measure to prevent or respond to VAWC had been implemented in the context of the pandemic. The most common types of measures were government-led or government-sponsored measures, of which at least one example was reported for 50 of the 53 member states and in territories Kosovo[1] and Greenland. NGO and civil society-led responses were reported at least once in 40 of the 53 member states and in the territory of Kosovo[1]. Table 1 displays the full break down of the types of measures across countries, with further details reported in Supplementary Table 2.

Most media reports used the term domestic violence, often without clarity as to whether the term was used to encompass both children and adults. Unless stated otherwise, in this report, the term domestic violence is interpreted to cover both women and children. Out of the 53 member states and two additional territories included in the study, all but one country reported measures to prevent or respond to 'domestic violence', 33 (60%) implemented measures to prevent or respond to VAW explicitly and 32 (58%) to VAC explicitly.

Government led or sponsored measures

The most frequently reported national or regional government measures were media and other information dissemination campaigns, reported in 39 member states, through various media channels. The Irish Department of Justice, for example, collaborated with frontline services to develop television, radio and social media advertisements highlighting their continued support for those subjected to abuse.[9] In Slovenia, the City of Ljubljana distributed leaflets with VAWC NGO contact numbers, along with the digital broadcasting of violence hotline numbers on public screens.[10]

Regarding high-level intersectoral approaches, governments at the national and regional level created government task forces to help prevent and respond to VAWC during COVID-19. In Belgium, the Brussels and Walloon governments created a task force in order to monitor shelters, assist actors in the field, rapidly identify emerging needs and spread information and awareness.[11] The government of Luxembourg created a crisis management system to mitigate the risk of increased VAWC.[12]

A number of countries dedicated specific funding for measures to prevent and respond to VAWC, including the United Kingdom, France, Israel, the Republic of Ireland, Italy, Sweden, and Kosovo[1].[11 13-18] The government of Iceland also included investment in efforts to combat domestic violence in their national financial aid package announcement.[19]

Various government guidance packages and policies were announced, either specifically for VAWC or with measures to respond to VAWC included, across member states (see Supplementary table 1 for details). This included the creation of a committee to examine the incidence of women killed during lockdown in Israel; or an interagency communication strategy on VAWC during the COVID-19 crisis in Georgia.[11 20]

[1] All references to Kosovo in this document should be understood to be in the context of the United Nations Security Council resolution 1244 (1999).

For peer review only

In terms of service coordination, multiple examples were found of government-led measures for VAWC, primarily supporting the maintenance and expansion of VAWC helplines and shelters. Methods varied from the introduction of new helplines numbers, such as text messaging numbers introduced in France and Israel, to the introduction of WhatsApp services in Spain.[11 14 21] The maintenance of shelters was also reported as a key priority for governments during this time, with measures taken to ensure they remained open or were expanded, for example, by providing them with hotels or additional accommodation.[22-28] A few governments also provided PPE and COVID-19 testing for staff and survivors and declared shelter staff as essential workers to exempt them from lockdown measures.[11 29-32]

In terms of moving VAWC resources such as psychosocial support and counselling online, 15 governments (14 member states plus Kosovo[1]) announced technology-based solutions, including a new government-led email address accessible to survivors and professionals in Portugal and the French online resource, stopblues.fr, that provides support and aids reporting.[11 33]

Pharmacies and supermarkets were also used to spread information; some governments sought to physically disseminate information such as numbers for VAWC helplines and relevant service providers, while other governments went further and implemented pop-up counselling centres in some supermarkets.[11 24 34] Pharmacies, specifically, were also encouraged by governments to participate in the European scheme using the 'Mask-19' code word, whereby pharmacy visitors could mention 'Mask-19' if they required help for domestic violence.[11 35-38]

Several member states reported working with the police to address the expected rise in VAWC during COVID-19. In the Republic of Ireland, the police service proactively contacted every previous survivor of domestic abuse known to them.[39] The government of Andorra created a video tutorial updating police officers on the VAWC guidelines protocol[11] and the Norwegian police implemented a comprehensive set of measures to react to changes in the levels of violence and ensure that police would focus on VAWC cases.[11] To ensure survivors could access required services, apps were identified for them to contact the police; in Czechia, the government disseminated the 'Bright Sky' app to allow survivors to contact support organisations and the police, and also to access advice and store evidence.[11]

Other measures focused on improving legal processes or provisions include the fast-tracking, prioritising and extension of legal processes surrounding cases of VAWC. In particular, in Croatia allowances were made for court deadlines missed by survivors due to COVID-19; while in Serbia, the High Court Council declared that despite the courts closing, domestic violence cases would continue to be processed.[11 40] In the Russian Federation, newspapers reported the introduction of mandatory reporting, obliging the police to investigate cases of VAWC even without an official request from the survivor.[41] In several other countries, governments introduced rules to ensure that in situations of abuse the perpetrator is evicted instead of the survivor.[11 42-44] In Kyrgyzstan, a bill was passed increasing detention of perpetrators of domestic violence to 48 hours, previously three hours.[45] Policies around releasing prisoners early or granting pardons under the pandemic situation excluded prisoners convicted on VAWC charges from release in several member states.[46-51]

For peer review only

Some government measures were targeted at improving the response to those experiencing violence. In many countries facing stricter lockdown rules, such as Italy, Spain and Kosovo[1], governments announced that the stay home orders and strict curfews did not apply to survivors who were seeking support.[42 43 52] Furthermore, in Malta and the Republic of Ireland, women and children known to be trapped in abusive homes were reportedly provided rent supplements.[53 54] Governments in Czechia, France and the United Kingdom also promoted the use of couriers and postmen to check in with survivors of abuse.[11 18 27]

In some cases, governments and NGOs jointly led measures to prevent and respond to VAWC. A technical group was formed in Montenegro within the European Union and UN Women-led regional programme to prepare guidance for institutions on how to deal with VAWC during COVID-19.[11] In Albania, the Women Forum Elbasan collaborated with state police to allow beneficiaries to be accompanied by police patrol, ensuring that they could access VAWC services despite the lack of transport available during the pandemic.[55] Furthermore, in Uzbekistan, a joint project with the United Nations Development Programme and the Ministry for Supporting Mahallas and Families prepared flyers that were distributed to pharmacies in Tashkent City and Tashkent region of Uzbekistan in order to reach vulnerable groups of the population and provide them with referral numbers in case of violence.[56] Measures that were not attributable to an organising body but picked up in the search included the printing of VAWC helpline numbers on milk bottles in Germany.[57] In Sweden, a popular landlord company distributed flyers with VAWC-related information to all its tenants.[57]

NGO and/or civil society led measures

Media campaigns and information dissemination were also one of the most frequently reported measure used by NGOs and civil organisations to prevent and respond to VAWC during COVID-19, reported across 24 Member States plus Kosovo[2]. Their strategies varied but were most often based around radio, television and social media. For example, the Union of Women Association of Heraklion in Greece, ensured a constant presence on TV shows, news channels and radio commercials to spread awareness of VAW during COVID-19.[38] Through social media, an online awareness campaign, Stopfisha, was launched in France as a response to the suspected rise in revenge porn as a result of lockdown to help find survivors of abuse and help them to report it.[58 59] As well as raising awareness and providing service contact details, social media was also used by NGOs and civil organisations to help those needing assistance regarding VAWC to seek help. For example, in Poland, a fictitious online cosmetic store was set up through Facebook where survivors of domestic violence could request help by pretending to order goods.[60]

Similar to the use of social media, NGOs and civil organisations in 24 member states plus Kosovo[1] used online methods and five member states developed apps in order to facilitate access to VAWC support services and/or to provide them with psychological and legal support through online platforms such as Zoom and Skype. NGOs across member states provided

[1] All references to Kosovo in this document should be understood to be in the context of the United Nations Security Council resolution 1244 (1999).
[2] All references to Kosovo in this document should be understood to be in the context of the United Nations Security Council resolution 1244 (1999).

services via Telegram, WhatsApp and Viber.[18 61-66] The NGO SPAVO in Cyprus purchased 35 smart watches for women facing domestic violence that had built-in safety alarms.[67]

The maintenance and expansion of helplines and helpline services was a key measure taken by NGOs and civil society groups across 19 member states, plus Kosovo[1]. NGO-led shelters in the Republic of Ireland expanded capacity by collaborating with Airbnb, in France a sports stadium was used and in Italy collaborations emerged with booking.com and also a former convent.[23 68 69] In the Republic of Moldova, to overcome government-imposed quarantine measures that meant shelters were unable to accept new residents, the NGO Promo-lex rented an apartment.[13] To ensure that helplines remained functional and could meet demand, measures included increasing the number of helpline volunteers, creating chat/SMS messaging options and expanding helpline hours.[11 55 70-75] New helplines were also set up by NGO and civil organisations in several members states.[11 74 76-80] In France the new helpline, "Don't Hit", was launched in April to provide counselling and specialist psychological assistance to perpetrators of violence.[11] Furthermore, systems were reportedly set up to allow helpline staff to work from home and PPE and/or COVID-19 testing were made available to VAWC centre staff in several countries.[11 55] The NGO 'CAM Firenze' in Italy organised fortnightly peer-support meetings to help staff with their emotional management and well-being during the pandemic.[72] In addition, one Belgian NGO set up open air face-to-face appointments, where survivors could receive support whilst on a socially-distant walk with centre staff.[38]

Similarly to the previous measures taken by governments to use pharmacies and supermarkets as help points for survivors, NGOs in North Macedonia, Italy and Greece also used them for the physical distribution of leaflets and posters with relevant VAWC service provision details.[38 55 81]

Other NGO and civil organisation measures conducted in only a few countries include proactive contacting and monitoring of both perpetrators (Luxembourg and Slovenia) and known survivors (Serbia, Republic of Moldova, Italy and Belgium)[11 38 55 72], food and/or hygiene packages distribution to vulnerable populations, including those affected by violence, in Malta, Naples (Italy), North Macedonia and Albania and financial and/or in-kind support to vulnerable groups in Bosnia and Herzegovina, Spain and Montenegro.[11 55 82-85] Other forms of support include the provision of free rail travel by a rail company in the United Kingdom (in partnership with the NGO Women's Aid). In Serbia, where public transport was abolished during the pandemic, multiple NGOs provided private transport for survivors to access their facilities.[55] Furthermore, in the United Kingdom, dentists were called on to update their guidelines for telephone assessments under lockdown to help them continue to assess the risk of VAWC in patients with facial injuries.[86]

Measures to prevent and respond to VAWC were also implemented by international organisations. In Kosovo[1], body cameras were provided by UN Women to the police's domestic violence department to ensure sufficient evidence was collected during call outs.[87] In Serbia, the United Nations Development Programme supported public prosecutors from eight prosecution districts to organise online multi-agency meetings to process cases of VAWC.[40] The All-Ukrainian Charity Foundation, UNICEF and the United Nations Foundations supported sociopsychological assistance mobile crews in the Ukraine.[88]

---

[1] All references to Kosovo in this document should be understood to be in the context of the United Nations Security Council resolution 1244 (1999).

For peer review only - http://bmjopen.bmj.com/site/about/guidelines.xhtml

Measures specifically to prevent violence against children

Implemented measures described above were often summarised under the headings of domestic violence, yet were more focused on addressing VAW rather than VAC explicitly. Only a few measures were identified that addressed VAC specifically, for example, the government of France set up services to allow children facing situations of violence to directly contact authorities via SMS.[89] In Ukraine, a chatbot was set up via Telegram to provide answers to common GBV-related questions and facilitate communication with state legal aid workers, this measure was reportedly popular amongst teen users.[90] In Sweden, the social media app 'Snapchat' was used by service providers to raise awareness and reach out to those aged 13 to 21 years who were potentially at risk of abuse. Further, in France, a children's aid charity partnered with gaming platform Fortnite to allow NGO volunteers to roam the virtual battle arenas; players could reach out to them privately to report abuse and ask for advice and assistance[91]. Those services all targeted older children and teenagers, and no reports could be found around measures addressing and preventing VAC among younger children.

DISCUSSION

This study provides an initial overview of some of the measures taken by the member states of the WHO European Region to prevent and respond to VAWC during the COVID-19 pandemic. Government responses were identified for 50 out of the 53 member states of the WHO European Region; NGOs and civil organisation-led measures were identified in 40 member states in the Region. The most frequently reported measures found in the study were media campaigns and other forms of information dissemination. Ensuring women and children are aware of the resources available to them and how the situation of the pandemic may affect their access to such resources is crucial and aligns with WHO recommendations. The high frequency of these measures may be due to political factors, and the fact that they can be rapidly arranged and implemented with a relatively low financial or resource burden. A WHO baseline assessment on health system responses to violence against women in the WHO European Region published in 2019 shows a very high proportion of WHO European member states have made national policy commitments to eliminate VAW and had national or sub-national multisectoral action plans to prevent and respond to VAW. This lays a good ground for information dissemination and media campaigns on this topic. The same assessment further found that a specific budget line for financing a health system response to VAW only existed in five European countries.

Media campaigns and information dissemination were some of the only reported *prevention* measures and existing evidence suggests that they are rarely effective on their own[92]. This is especially pressing given the long-term impact of the pandemic on poverty and economic well-being, both factors that are known to be linked to VAWC[93]. The majority of the results of this study pertain to *response* measures. As very few preventative measures were identified by this report, it suggests that they were not only rarely implemented, but that existing prevention measures may have also been halted during the pandemic. This is critical as, without the continuation of prevention methods, the pandemic will have far-reaching and long-term impacts on VAWC and due to this, the measures taken to respond to VAWC during this time should be continued long into the future to mitigate these long-term impacts.

For peer review only

The second most reported group of measures taken by governments pertain to another response measure, service coordination for survivors; in particular, the maintenance and expansion of shelters and helplines and online services for women and children facing violence. This is in line with the UN recommendations that shelters should be classified as essential services during COVID-19 and that women should have access to safe ways to seek support.[94] The need to expand or strengthen VAWC services during a pandemic could be dependent on how well these services were already supported prior to the pandemic, as well as the nature of the lock-down measures. Several countries pledged additional funds to civil society organisations to respond to the increased service needs, but our findings currently do not reveal if countries also allocated investments to strengthen public health systems service provision. Some countries took measures to facilitate physical access to services, while other moved services online or through apps.

Many of the measures taken by governments and NGO/civil organisations mentioned the continuation of counselling and psychosocial support, predominantly through the move to online methods of communication, which follows the WHO recommendation of ensuring women-centred interventions for survivors.[92] However, measures ensuring that services are available and accessible online were more often the work of NGOs and civil society organisations than of governments. Besides the movement of resources online, our study found other interesting examples of where certain measures were more often reported as being NGO and civil organisation-led than government-led. For example, the provision of financial and good packages. Such provisions included money, food and hygiene-related items and were most often provided to groups identified by the NGOs as vulnerable. Although not well represented in the results of this study, vulnerable groups will have likely been some of the worst affected populations during the pandemic, not only regarding VAWC. The fact that support to vulnerable groups was most often reported as NGOs/civil organisation-led highlights how such populations are often under-represented in government-led responses and the important role civil society has in filling these gaps. Non-governmental/civil society organisations also often had to mitigate the negative impact of government-led pandemic responses. One example being the provision of transport in several member states where, in the absence of government allowances for certain groups to break lockdown rules, civil society organisations had to provide alternative means of transport. Furthermore, NGOs and civil organisations were also frequently reported as leading advocacy campaigns aimed at raising awareness to VAWC in the context of the pandemic and demanding action. This highlights civil society as a crucial force for focusing attention to human rights issues and holding governments accountable.

From the results of this study, it is clear that measures identified to prevent and respond to VAWC were predominantly focused on VAW. The study did not identify any school-based measures and very few parenting programmes for the prevention and response to VAC. School-based interventions and parenting programmes are known to be effective in the prevention and reduction of VAC;[92 95] however, with widespread school closures across Europe, school-based interventions were not feasible. Further, with the current additional burden of home-schooling placed upon parents during COVID-19, the possibility of conducting parenting programmes during this time was low. Schools also provide a crucial pathway for the identification and response to VAC and therefore, with schools closed, it is likely that the majority of child abuse will have gone unreported during this time. As schools across many of the member states have now re-opened, this will be a crucial time to ensure that children are offered the necessary support and services that they may not have had access to under the pandemic restrictions. Furthermore, it is vital to consider the long-term impact that the pandemic will have had on children who have spent lockdown at home with abusive family members. VAC and VAW are both forms of domestic

For peer review only

violence and share many overlapping risk factors, however, prevention and response measures for the two types of violence can differ greatly, and so it is important that the need to address each separately is not overlooked when both are grouped under the term 'domestic violence'. The analysis most often only identified measures to address VAWC among the general population, not considering those who are particularly vulnerable to violence, such as migrant women, sex workers, or low-income families, who might also be affected more by COVID-19 restrictions.

To contextualise the results, there are some limitations of this study that should be considered. Firstly, the attention of media reports is often biased towards new and innovative strategies rather than the actions taken to maintain existing responses and systems. Therefore, countries with strong existing frameworks for VAWC prevention, who did not need to rely on novel responses, are very likely to be underrepresented in this data. Secondly, the use of only English or Russian search terms will have excluded reports in other European languages. Due to this, some countries will be overrepresented in these results, particularly the United Kingdom, Russian Federation and the Republic of Ireland. This varied representation, combined with the focus on innovative responses, means that a lack of data reported for a country should not be interpreted as a country's lack of action to protect women and children from violence during COVID-19. Similarly, due to the lack of evaluation of the presented measures, the frequency in which measures are reported here is not an indication of their effectiveness. For example, Greenland was the only jurisdiction that our study found to have enforced a regional alcohol ban for the specific purpose of preventing VAC, but due to the wealth of evidence linking alcohol consumption to VAWC, it is likely that this measure would have had a positive effect on the reduction of violence.[96 97] Furthermore, the newspaper articles do not give any indication at this stage on whether the measures had the proposed positive effect, had any adverse side effects and what level of training and support is needed to implement them. For example, using postmen to check on women at risk of VAW would require substantial training in VAW, being non-judgemental, ethics and safety. Finally, the search strategy only focused on reports explicitly referring to VAWC, thereby ignoring upstream preventative measures such as parental support, education and childcare provision that are not always mentioned in the context of VAC. This may explain the under-representation of child-focused responses in our results. Similarly, focusing only on reports that referred to violence means that many service-based measures were not widely represented in our results, such as measures to ensure access to sexual and reproductive health services, continued abortion care and access to HIV care and STI prophylaxis, all key aspects of clinical care when responding to VAW.[98]

CONCLUSION

These results provide evidence that a diverse set of measures were taken by European governments and civil society organisations to maintain and expand VAWC service provision during the first months of the pandemic. While it is clear that the COVID-19 context has led to an increased focus on VAWC, and that an overwhelming majority of countries have taken some kind of action, further research is needed on the impact of these actions and what can be learned from the past couple of months in order to "build back better". For some women and children, the living situation during lockdown is, unfortunately, not too dissimilar to pre-pandemic life living with an abuser. Therefore, rather than suggesting the implementation of the outlined emergency measures alone, governments should be encouraged to reflect on the gaps in existing national VAWC response frameworks in their countries; in particular, ensuring greater emphasis on VAC within

For peer review only

measures to prevent and respond to VAWC. So, while we should celebrate the ability of governments, NGOs and civil society organisations to rapidly adapt under pressure, the responsibility should now be on governments to develop stronger baseline support systems to ensure that the responsibility to protect women and children does not fall on NGOs and civil society as the world rebuilds from COVID-19. Further research is also needed to understand how the mental health impacts of the pandemic, along with the wider disruptions to service provision and access to work and education will affect VAWC in the long-term.

For peer review only

REFERENCES

1. García-Moreno C, Stöckl H. Protection of sexual and reproductive health rights: addressing violence against women. *International Journal of Gynecology & Obstetrics* 2009;106(2):144-47.
2. Devries KM, Mak JY, Garcia-Moreno C, et al. The global prevalence of intimate partner violence against women. *Science* 2013;340(6140):1527-28.
3. Hillis S, Mercy J, Amobi A, et al. Global prevalence of past-year violence against children: a systematic review and minimum estimates. *Pediatrics* 2016;137(3)
4. Krug EG, Mercy JA, Dahlberg LL, et al. The world report on violence and health. *The lancet* 2002;360(9339):1083-88.
5. Cluver L, Lachman JM, Sherr L, et al. Parenting in a time of COVID-19. 2020
6. Peterman A, Potts A, O'Donnell M, et al. Pandemics and violence against women and children. *Center for Global Development working paper* 2020;528
7. Mlambo-Ngcuka P. Violence against women and girls: The shadow pandemic. *UN Women* 2020
8. World Health Organization. Addressing violence against children, women and older people during the COVID-19 pandemic: Key actions: World Health Organization, 2020.
9. Hilliard M. Efforts to help trapped domestic abuse victims. *Irish Times* 2020 2020 Apr 16;9.
10. Novak P. Ljubljana – Domestic violence awareness 2020 [Available from: https://covidnews.eurocities.eu/2020/05/06/ljubljana-domestic-violence-awareness/.
11. Council of Europe. Promoting and protecting women's rights at national level 2020 [Available from: https://www.coe.int/en/web/genderequality/promoting-and-protecting-women-s-rights.
12. The Luxembourg Chronicle. Police Respond to 243 Domestic Violence Cases during COVID-19 Crisis. *The Luxembourg Chronicle* 2020.
13. Bami XD, Nedim; Stojanovic, Milica; Marusic, Sinisa Jakov; Necsutu, Madalin; Kajosevic, Samir; Vladisavljevic, Anja; Barberá, Marcel Gascón; Todorov, Svetoslav COVID-19 and Domestic Abuse: When Home is not the Safest Place. *Balkan Insight* 2020.
14. Bisserbe NL, Pietro. As Coronavirus Piles Pressure on Families, Domestic Violence Concerns Surge. *The Wall Street Journal* 2020.
15. Horne M. BRITAIN'S MOST TRUSTED NATIONAL NEWSPAPER CORONAVIRUS SUMMARY: GLOBAL CONFIRMED CASES UK CONFIRMED CASES 4.9M 323,017 248,818 35,341 GLOBAL DEATHS UK DEATHS [SCOT REGION]. *The Times* 2020 May 20;2.
16. Mahar M. Increased Domestic Violence after COVID-19 Lockdown. *Daily Times* 2020.
17. Times of Israel. Thousands rally in Tel Aviv against violence towards women. *The Times of Israel* 2020 2020 Jun 01.
18. UN Women. COVID-19 and essential services provision for survivors of violence against women and girls. EVAW COVID-19 briefs, 2020:11.
19. Askham P. COVID-19 In Iceland: Tenth Death, New Government Package Announced. *The Reykjavík Grapevine* 2020.
20. Cashman G. The day has come. *Jerusalem Post* 2020 2020 May 24;12.
21. Winer S. Welfare Ministry: 4 suicides due to domestic violence amid coronavirus crisis. *The Times of Israel* 2020 05/03/2020
22. Abels R. Als een Française om 'masker 19' vraagt, weet de apotheker dat hij de politie moet bellen. *Trouw* 2020.
23. Cook C. Marseille club opens to victims of domestic violence. *Anadolu Agency : AA* 2020 2020 Apr 21.
24. Dombey D, Abboud L. Victims of lockdown abuse struggle to speak out: Domestic violence. Social cost Spanish and French officials fear that despite extra schemes for women many could not get help [Asia Region]. *Financial Times* 2020 2020 May 27;2.
25. Evers F. Extra Vlaams geld tegen huishoudelijk geweld. *DeMorgen* 2020.
26. Khan SR. Are we ready for shadow pandemic? *New Age* 2020.
27. Мухамеджанов Б. НИКАКИХ ПРОБЛЕМ: ВЛАСТЬ И ДОМАШНЕЕ НАСИЛИЕ В ЭПОХУ ПАНДЕМИИ. *Republic (Slon)* 2020.
28. Расулова О. «Пандемия внутри пандемии». Как карантин в Украине влияет на домашнее насилие. *Hromadske* 2020.
29. Gilbert D. How many coronavirus tests are being done in the UK? *Telegraphcouk* 2020 2020 May 20.
30. Kippenberg J. Millions of Children Threatened by Violence Risk Being Forgotten Amid Coronavirus Lockdowns. *Euronews* 2020.
31. Peter M. Coronavirus: Four new cases reported on Tuesday (updated). *Cyprus Mail* 2020 2020 May 05.

For peer review only

32. Wilkinson I. Spain forced to tighten its lockdown [Edition 2]. *The Times* 2020 2020 Mar 31;14.
33. Mengin A, Allé MC, Rolling J, et al. Psychopathological consequences of confinement. *Encephale* 2020;46(3S):S43-S52. doi: 10.1016/j.encep.2020.04.007 [published Online First: 2020/04/22]
34. Hymas C. Safe spaces should be set up in supermarkets for domestic abuse victims, Home Affairs committee advises. *Telegraphcouk* 2020 2020 Apr 27.
35. ANI. In COVID-19 lockdown, US reports surge in domestic violence. *Times of India* 2020.
36. Reuters News Service. UK plans codeword scheme to tackle rise in domestic abuse. *Cyprus Mail* 2020 2020 May 21.
37. Thompson R. World: Trainee doctor slayed as domestic violence reports jump amid lockdown. *Asia News Monitor* 2020 04/20/2020.
38. Conference: Perpetrator Work During COVID-19. Perpetrator Work During COVID-19; 2020; Online.
39. Begley I. Lockdown for victims of domestic abuse is a ... LIVING HELL: coronavirus crisis [Eire Region]. *Daily Mail* 2020 2020 Apr 11;10.
40. UNDP Serbia. COVID-19: UNDP urges swift action to address violence against women and girls during pandemic 2020 [Available from: https://www.rs.undp.org/content/serbia/en/home/presscenter/articles/2020/undp-poziva-na-hitnu-akciju-za-reavanje-nasilja-nad-enama-i-devo.html.
41. BBC Monitoring. Russia: Domestic violence in spotlight amid Covid-19 outbreak. *BBC Monitoring Former Soviet Union* 2020.
42. Le Page I. Fears of domestic violence rise as millions confined over virus. *CTV News* 2020.
43. Graham-Harrison EG, Angela; Smith, Helena; Ford, Liz. Lockdowns around the world bring rise in domestic violence. *The Guardian* 2020.
44. Dellanna A. France: France approves new measures to fight the Covid-19 'catastrophe' as it prepares to ease lockdown. *Asia News Monitor* 2020 2020 May 11.
45. Kloop. Kyrgyzstan acts to toughen domestic violence law. *BBC Monitoring Central Asia* 2020 2020 May 03.
46. Agence France Presse. N.Ireland to release around 200 prisoners to stem COVID-19 spread. *AFP International Text Wire in English* 2020 2020 Mar 30.
47. Daragahi B. Coronavirus: Turkey to free thousands of convicted criminals but keep journalists behind bars. *Independent* 2020.
48. Financial Mirror. COVID19: Cyprus takes action to decongest prisons. *Financial Mirror* 2020 2020 Mar 31.
49. Gradaigh PO. French warships to back up far-flung territories in virus fight. *DPA International (English)* 2020 2020 Mar 25.
50. Xinhua. Portugal to release up to 2,000 prisoners over COVID-19 pandemic. *Xinhua News Agency - CEIS* 2020 2020 Apr 10.
51. Zonshine I. Israel to release hundreds of prisoners to curb spread of coronavirus. *The Jerusalem Post (Online)* 2020 [published Online First: English ed.]
52. UN Women. Temporary shelters set up to quarantine safely domestic violence survivors in Kosovo 2020 [Available from: https://eca.unwomen.org/en/news/stories/2020/5/temporary-shelters-set-up-to-quarantine-safely-domestic-violence-survivors.
53. Bonnici O. A better tomorrow. *The Malta Independent* 2020 2020 Jun 12.
54. McMahon A, Phelan C. Rent relief for abuse victims. *The Daily Mirror* 2020 2020 Jun 15;6.
55. UN Women. Impact of the COVID-19 pandemic on specialist services for victims and survivors of violence in the Western Balkans and Turkey: A proposal for addressing the needs: UN Women, 2020:156.
56. UNDP Uzbekistan. Reducing domestic violence caused by the COVID-19 lockdown in Uzbekistan, 2020.
57. Exchange with cities on the impact of the COVID-19 crisis on women. Exchange with cities on the impact of the COVID-19 crisis on women; 2020; Online.
58. Davies S. French revenge porn activists mount 'virtual raids'. *Reuters* 2020.
59. Rivers D. Revenge porn soars across Europe during lockdown as cases double in Britain. *Daily Star (Online)* 2020 2020 May 06.
60. Bretan J. New domestic violence law in Poland to immediately remove perpetrators from the home. *Notes from Poland* 2020.
61. Andreou E. Coronavirus: online event this month for women in danger of domestic violence. *Cyrpus Mail* 2020.
62. Percannella A. ‘A double layer of isolation’: domestic abuse survivors struggling to call for help. *The Independent (Daily Edition)* 2020 2020 Apr 23;27.

For peer review only

63. Bouvier A. New Fortnite mission: Reaching out to abused children. *AFP International Text Wire in English* 2020 2020 Jun 16.
64. EUAM Ukraine. Domestic violence and quarantine: situation becomes more grave under lockdown, 2020.
65. Lepeska D. Turkish women's rights movement fights back as COVID-19 spurs violence. *Ahval news* 2020 2020 May 28.
66. Ukrinform. МВД запустило в Telegram чат-бот для противодействия домашнему насилию. *Ukrinform* 2020.
67. Agapiou G. Coronavirus: police say domestic violence 'not up significantly' because of lockdown. *Cyprus Mail* 2020 2020 Apr 24.
68. Davies SaB, Emma. Europe braces for domestic abuse surge 'perfect storm' amid coronavirus lockdown. *Reuters* 2020 2020 Mar 26.
69. Mangan I. Airbnb offers free stays to aid families [Eire Region]. *The Daily Mirror* 2020.
70. Agapiou G. Coronavirus: Skype seminar Thursday to discuss domestic violence. *Cyprus Mail* 2020 04/29/2020
71. Armellini A. Italy: Coronavirus lockdown raises domestic violence risks for women. *DPA International (English)* 2020 03/20/2020.
72. WWP EN. Interview with CAM Firenze. Online, 2020.
73. Gaunt M. Trauma' fear for children in lockdown. *The Yorkshire Post* 2020 2020 Apr 27;5.
74. Meagher JC, Ellen. 'There's no respite': the devastating impact of abuse during lockdown. *Irish Independent* 2020 05/09/

2020 May 09;6.
75. Shtotland I. Domestic violence in the time of corona. *Jerusalem Post* 2020 04/24/2020;22.
76. Times of Israel. Crisis hotline says thousands of Israelis calling over virus, isolation worries. *The Times of Israel* 2020 2020 Mar 14.
77. OSCE. Championing Gender in Uzbekistan: Interview with John MacGregor, OSCE Project Co-ordinator in Uzbekistan, 2020.
78. Sputnik News. Пандемия домашнего насилия: как бороться с жестокостью в семье. *Sputnik UZ* 2020.
79. Захваткина А. По-соседски: как защищают права женщин в России, Таджикистане и на Украине. *Agency of Social Information* 2020.
80. Newey SR, L; Davies, G; Gartner, A; Global Health Security Team,. Government releases more home test kits for key workers after slots run out in two minutes. *The Telegraph* 2020.
81. Grierson J. Calls for funds to house domestic violence victims during Covid-19 outbreak. *The Guardian* 2020.
82. Briguglio M. A social pact for Worker's Day – Michael Briguglio. *The Malta Independent* 2020 2020 Apr 30.
83. Jones S. 'The situation is critical': coronavirus crisis agony of Spain's poor. *The Guardian (Online)* 2020
84. UN Women. Press Release: UN Women takes initiative to ensure that women and families hit hardest by the COVID-19 crisis are not left behind in North Macedonia. Skopje, 2020.
85. Tondo L. Sex traffickers left thousands of women to starve during Italy lockdown. *The Guardian* 2020.
86. Coulthard P, Hutchison I, Bell JA, et al. COVID-19, domestic violence and abuse, and urgent dental and oral and maxillofacial surgery care. *British dental journal* 2020;228(12):923-26.
87. Webinar: Covid-19 and domestic violence against women; 2020 20 July; Online.
88. BBC Monitoring. Ukraine: Donetsk Region media highlights 13-19 Jun 20. *BBC Monitoring Former Soviet Union* 2020.
89. PTI. Calls to France's child-abuse hotline surge during lockdown. *AFP International Text Wire in English* 2020 2020 Apr 09.
90. UNFPA. Silent solutions available to quarantined survivors of domestic violence 2020 [Available from: https://www.unfpa.org/news/silent-solutions-available-quarantined-survivors-domestic-violence.
91. Agence France-Presse. New Fortnite Mission: Reaching Out to Abused Children. *NDTV* 2020 17 June 2020.
92. Ellsberg M, Arango DJ, Morton M, et al. Prevention of violence against women and girls: what does the evidence say? *The Lancet* 2015;385(9977):1555-66.
93. Stöckl H, Heise L, Watts C. Factors associated with violence by a current partner in a nationally representative sample of German women. *Sociology of health & illness* 2011;33(5):694-709.
94. Guterres A. 'Make The Prevention And Redress Of Violence Against Women A Key Part Of National Response Plans For COVID-19'. *United Nations* 2020.
95. World Health Organization. INSPIRE: Seven strategies for ending violence against children: World Health Organization 2016.
96. World Health Organization. Interpersonal violence and alcohol. *World Health Organisation, Geneva* 2006

97. Agence France Presse. Greenland bans booze in capital over virus fears. *AFP International Text Wire in English* 2020 03/29/2020.
98. World Health Organization. Responding to intimate partner violence and sexual violence against women: WHO clinical and policy guidelines: World Health Organization 2013.

For peer review only

Table 1: Reported measures in WHO European Member States, including territories Kosovo[1] and Greenland (categorised by type, number of countries and percentage of countries)

Implemented Measure	Number of countries in which measure, was reported, overall (%)	Number of countries in which measure(s) were reported as led/sponsored by gov. (national)	Number of countries in which measure(s) were reported as led/sponsored by gov. (regional)	Number of countries in which measure(s) were reported as led by NGO/Civil society	Number of countries in which measure(s) were reported as led by NGO/Civil society and gov. in partnership	Number of countries where leading body was unclear
**Response services**						
Helpline expansion/maintain	43 (78.2)	33	3	20	2	17
Shelter expansion/maintain	38 (69.1)	25	8	14	6	12
Move resources online	38 (69.1)	15	2	25	4	10
VAWC App	13 (23.6)	11	0	5	0	2
Financial/goods support	13 (23.6)	5	1	9	1	1
Extra funding for NGOs	13 (23.6)	11	1	3	0	0
Proactive contact with survivors	11 (20.0)	8	2	3	0	0
Emergency mobile teams	3 (5.5)	1	0	1	1	0
Monitor past perpetrators	3 (5.5)	0	0	3	0	1
EU funding earmarked for VAWC services	2 (3.6)	2	0	0	0	0
Testing for survivors	1 (1.8)	1	0	0	0	0
Open air F2F appointments	1 (1.8)	0	0	1	0	0
**Awareness and outreach**						
Media campaign/Info dissemination	48 (87.3)	37	6	25	4	8
Official guidance/policy	30 (54.5)	26	0	0	9	0
Advocacy	11 (20.0)	3	2	8	0	0
Creation of taskforce	7 (12.7)	6	1	0	1	0
Community/corporate Fundraising for VAWC services	3 (5.5)	0	0	3	0	0
**Legal support for survivors**						
Fast track/prioritise/extend legal processes	15 (27.3)	13	0	2	1	3
Exempt from lockdown measures*	13 (23.6)	11	1	1	0	2
Eviction of perpetrator	5 (9.1)	3	0	0	0	3
Police prioritise DV cases	3 (5.5)	3	0	0	0	0
Alcohol ban	1 (1.8)	0	1	0	0	0
**Coordination with other services**						
Pharmacy help point	14 (25.5)	3	3	6	1	8
Raise police awareness	9 (16.4)	8	0	1	1	0
Supermarket/shop help point	5 (9.1)	2	0	3	0	2
Ensure provision of existing SRH services	5 (9.1)	2	1	0	0	2
Childcare provision	4 (7.3)	1	1	1	0	1
Postman check in	3 (5.5)	3	1	1	0	1
Free transport	2 (3.6)	0	0	2	0	0
Police codeword	1 (1.8)	1	0	0	0	0
PPE for Police	1 (1.8)	1	0	0	0	0
Dentist guidelines for telephone assessment	1 (1.8)	0	0	1	0	0
Medical care for refugees	1 (1.8)	1	0	0	0	0
Other service coordination	3 (5.5)	0	0	2	2	0
**Strengthen capacity/protection for professionals**						
PPE/Testing for VAWC staff	11 (20.0)	7	0	5	0	1
Other support for VAWC centre staff	3 (5.5)	1	0	2	1	0

[1] All references to Kosovo in this document should be understood to be in the context of the United Nations Security Council resolution 1244 (1999).

For peer review only

** 'Exempt from lockdown measures' refers to a situation where those facing violence within the home were exempt from the strict lockdown measures imposed in their local area, for example, being exempt from curfews or being allowed to use public transport to access support services*

For peer review only

Supplementary Table 1: Number of excluded studies and reason for exclusion from the ProQuest search output

**Exclusion Reason**	**Number Excluded**
Total found from search	2,343
Total full text search	1,610
Not related to either VAW/VAC or COVID-19	329
Related to VAW/VAC but not related to COVID-19	298
Related to COVID-19 but not related to VAC/VAW	208
Insufficient Information	242
Related to VAW/VAC and COVID-19 but did not include any public health responses	252
Not relevant countries (not WHO European Member States or territories Kosovo[1] or Greenland)	74
Duplicates	47
Individual case studies	3
No access to the full text	1
Wrong date	1
**Total excluded**	**1,455**
**Total included**	**155**

[1] All references to Kosovo in this document should be understood to be in the context of the United Nations Security Council resolution 1244 (1999)

For peer review only - http://bmjopen.bmj.com/site/about/guidelines.xhtml

Supplementary Table 2: Type of measures reported by each of the WHO European Member States, including territories Kosovo[1] and Greenland

	Albania	Andorra	Armenia	Austria	Azerbaijan	Belarus	Belgium	Bosnia and Herzegovina	Bulgaria	Croatia	Cyprus	Czechia	Denmark	Estonia	Finland	France	Georgia	Germany	Greece	Greenland	Hungary	Iceland	Ireland	Israel	Italy	Kazakhstan	Kosovo	Kyrgyzstan	Latvia	Lithuania	Luxembourg	Malta	Monaco	Montenegro	Netherlands	North Macedonia	Norway	Poland	Portugal	Republic of Moldova	Romania	Russian Federation	San Marino	Serbia	Slovakia	Slovenia	Spain	Sweden	Switzerland	Tajikistan	Turkey	Turkmenistan	Ukraine	United Kingdom	Uzbekistan
Innovative Response	*Y/N*																																																						
Media campaign/Info dissemination	Y	Y	Y	Y	Y	Y	Y	Y	N	Y	Y	Y	N	Y	Y	Y	Y	Y	Y	N	N	N	Y	Y	Y	Y	Y	Y	Y	N	Y	Y	Y	Y	Y	Y	Y	Y	Y	N	Y	N	Y	Y	Y	Y	Y	Y	Y	N	Y	N	Y	Y	Y
Helpline expansion/maintain	Y	Y	N	N	Y	N	Y	Y	Y	Y	Y	N	Y	N	Y	Y	Y	Y	Y	N	N	N	Y	Y	Y	N	Y	N	N	Y	Y	Y	Y	Y	N	Y	Y	N	Y	Y	Y	Y	Y	Y	N	Y	Y	Y	N	N	Y	N	Y	Y	Y
Shelter expansion/maintain	Y	N	Y	N	N	N	Y	Y	N	Y	Y	N	Y	Y	Y	Y	Y	Y	Y	N	N	Y	Y	Y	Y	Y	Y	Y	Y	N	N	Y	N	N	Y	N	Y	Y	Y	Y	N	Y	N	Y	Y	Y	Y	N	Y	N	Y	N	Y	N	Y
Move resources online	Y	Y	N	Y	Y	N	Y	Y	N	Y	Y	N	Y	Y	N	Y	N	Y	Y	N	Y	N	Y	N	Y	N	Y	Y	Y	N	Y	Y	Y	Y	N	Y	N	Y	Y	Y	N	Y	N	Y	N	N	Y	N	N	N	Y	N	Y	Y	Y
Government guidance/policy	Y	N	N	Y	N	N	N	N	N	N	Y	N	N	N	N	Y	Y	Y	N	N	N	N	Y	Y	Y	Y	N	Y	N	Y	N	N	N	Y	N	N	N	Y	Y	Y	Y	Y	N	N	Y	N	Y	N	N	N	Y	N	Y	Y	N
App	N	N	N	N	N	N	N	N	N	N	Y	Y	N	N	N	N	N	N	N	N	N	N	N	N	Y	N	N	N	N	N	N	Y	N	Y	N	N	N	N	N	N	N	Y	Y	N	N	N	Y	N	N	N	Y	N	Y	Y	Y
Financial/goods support	Y	N	Y	N	Y	N	N	Y	N	N	N	N	N	Y	N	N	N	N	N	N	N	N	Y	N	Y	N	N	N	N	N	N	Y	N	Y	N	Y	N	N	N	N	N	N	N	N	N	N	Y	N	N	N	N	N	N	N	N
Pharmacy help point	N	N	N	N	N	N	Y	N	N	N	N	N	N	N	N	Y	N	Y	Y	N	N	N	Y	N	Y	N	Y	N	N	N	N	N	Y	N	N	N	Y	N	N	N	N	N	N	Y	N	Y	Y	N	N	N	N	N	N	N	N
Extra funding for NGOs	N	N	N	N	N	N	Y	Y	N	N	N	N	N	N	N	Y	N	N	N	N	N	Y	Y	N	Y	N	Y	N	N	N	N	N	N	N	N	N	N	N	N	N	N	N	N	Y	N	N	N	Y	N	N	N	N	N	Y	N
Advocacy	N	N	N	N	N	N	N	N	Y	N	Y	N	N	N	N	N	N	N	N	N	N	N	Y	Y	N	Y	N	Y	Y	N	N	Y	N	N	N	N	N	N	N	N	N	N	N	N	N	N	N	N	N	N	N	N	Y	N	Y
Loosen lockdown measures	Y	N	N	Y	N	N	N	N	N	N	N	N	N	N	N	N	Y	N	N	N	N	N	Y	N	Y	N	Y	N	N	N	N	Y	N	Y	Y	N	N	N	N	N	N	N	N	N	N	N	Y	N	N	N	N	N	N	Y	Y
Fast track/prioritise/extend legal processes	Y	N	N	Y	N	N	Y	N	N	Y	Y	N	N	N	N	Y	N	N	N	N	N	N	Y	N	N	N	N	Y	N	N	N	N	N	Y	N	N	N	N	N	N	N	N	N	Y	N	N	N	N	N	N	N	N	N	N	Y
Proactive contact with survivors	N	N	N	N	N	N	Y	N	N	N	N	N	N	N	N	Y	N	N	N	N	N	N	Y	N	Y	N	N	N	N	Y	N	N	N	N	N	N	N	Y	N	Y	N	N	N	Y	N	N	Y	N	N	N	N	N	N	N	N
Eviction of perpetrator	N	N	N	Y	N	N	N	N	N	N	N	Y	N	N	N	Y	N	N	N	N	N	N	N	N	Y	N	N	N	N	N	N	N	N	Y	N	N	N	N	N	N	N	N	N	N	N	N	N	N	N	N	N	N	N	N	N
Creation of taskforce	N	N	N	N	N	N	Y	N	N	N	N	N	N	N	N	N	N	N	N	N	N	N	Y	N	N	N	N	N	N	N	Y	N	N	Y	N	N	N	N	N	N	N	N	N	N	N	N	N	Y	Y	N	N	N	N	N	N
Raise police awarness	Y	Y	N	N	N	N	Y	N	N	N	N	N	N	N	Y	N	N	N	N	N	N	N	N	N	N	N	Y	N	N	N	N	N	N	N	N	N	Y	N	N	N	N	N	N	N	N	N	N	N	N	N	N	N	Y	N	N
PPE/Testing for VAWC staff	N	N	N	N	Y	N	N	N	N	N	N	N	N	N	N	N	N	N	N	N	N	N	Y	N	N	N	N	N	N	N	N	Y	N	N	N	N	N	N	N	Y	N	N	N	N	Y	N	N	N	N	N	N	N	N	Y	N
Childcare provision	N	N	N	N	N	N	N	N	N	N	N	N	N	Y	N	N	N	N	N	N	N	N	N	N	N	N	N	N	N	N	Y	N	N	N	N	N	N	N	N	N	N	N	N	N	N	Y	N	N	N	N	N	N	Y	N	N
Ensure provision of existing SRH services	N	N	N	N	N	N	N	N	N	N	N	N	N	N	N	Y	N	N	N	N	N	N	N	N	N	N	N	N	N	N	N	N	N	N	Y	N	N	N	N	N	N	N	N	N	N	N	N	N	N	N	N	N	N	Y	N
Postman checkin	N	N	N	N	N	N	N	N	N	N	N	Y	N	N	N	Y	N	N	N	N	N	N	N	N	N	N	N	N	N	N	N	N	N	N	N	N	N	N	N	N	N	N	N	N	N	N	N	N	N	N	N	N	N	Y	N
Other support for VAWC centre staff	N	N	N	N	N	N	N	N	N	N	N	N	N	N	N	N	N	N	N	N	N	N	N	N	Y	N	N	N	N	N	N	N	N	N	N	N	N	N	N	N	N	N	N	Y	N	N	N	N	N	N	N	N	N	N	N
Police prioritise DV cases	N	N	N	N	N	N	N	N	N	N	N	N	N	N	N	N	N	N	N	N	N	N	Y	N	N	N	N	N	N	N	N	N	N	N	N	N	N	N	N	N	N	N	N	N	N	N	N	N	Y	N	N	N	N	Y	N
Supermarket/shop help point	N	N	N	N	N	N	N	N	N	N	N	N	N	N	N	Y	N	N	N	N	N	N	N	N	N	N	Y	N	N	N	N	N	N	N	N	N	N	N	N	N	N	N	N	N	N	N	N	N	N	N	N	N	N	Y	N
Service coordination	Y	N	N	N	N	N	N	N	N	N	N	N	N	N	N	N	N	N	Y	N	N	N	N	N	N	Y	N	N	N	N	N	N	N	N	N	N	N	N	N	N	N	N	N	N	N	N	N	N	N	N	N	N	N	N	N
Community/corporate Fundraising	N	N	N	N	N	N	N	N	N	N	N	N	N	N	N	N	N	N	N	N	N	N	Y	N	N	N	N	N	N	N	N	N	N	N	N	N	N	N	N	N	N	Y	N	N	N	N	N	N	N	N	N	N	N	Y	N
Emergency mobile teams	N	N	N	N	N	N	N	N	N	N	N	N	N	N	N	N	N	N	N	N	N	N	N	N	N	N	N	Y	N	N	N	N	N	N	N	N	N	N	N	N	N	N	N	Y	N	N	N	N	N	N	N	N	Y	N	N
Social media codeword	N	N	N	N	N	N	N	N	N	N	N	N	N	N	N	N	N	N	N	N	N	N	N	N	Y	N	N	N	N	N	N	N	N	N	N	N	N	N	N	N	N	N	N	N	N	N	N	N	N	N	N	N	Y	N	N
Free transport	N	N	N	N	N	N	N	N	N	N	N	N	N	N	N	N	N	N	N	N	N	N	N	N	N	N	N	N	N	N	N	N	N	N	N	N	N	N	N	N	N	N	N	Y	N	N	N	N	N	N	N	N	N	Y	N
EU funding	N	N	N	N	N	N	N	N	N	N	N	N	N	N	N	N	N	N	N	N	N	N	N	N	N	N	N	Y	N	N	N	N	N	N	N	N	N	N	N	N	N	Y	N	N	N	N	N	N	N	N	N	N	N	N	N
Monitor past perpetrators	N	N	N	N	N	N	N	N	N	N	N	N	N	N	N	N	N	N	N	N	N	N	N	N	N	N	N	N	N	N	Y	N	N	N	N	N	N	N	N	N	N	N	N	N	N	Y	N	N	N	N	N	N	N	N	N
Testing for survivors	N	N	N	N	N	N	N	N	N	N	Y	N	N	N	N	N	N	N	N	N	N	N	N	N	N	N	N	N	N	N	N	N	N	N	N	N	N	N	N	N	N	N	N	N	N	N	N	N	N	N	N	N	N	N	N
Police codeword	N	N	N	N	N	N	N	N	N	N	N	N	N	N	N	N	N	N	N	N	N	N	N	N	N	Y	N	N	N	N	N	N	N	N	N	N	N	N	N	N	N	N	N	N	N	N	N	N	N	N	N	N	N	N	N
PPE for Police	N	N	N	N	N	N	N	N	N	N	N	N	N	N	N	N	N	N	N	N	N	N	N	N	N	N	N	N	N	N	N	Y	N	N	N	N	N	N	N	N	N	N	N	N	N	N	N	N	N	N	N	N	N	N	N
Open air F2F appointments	N	N	N	N	N	N	Y	N	N	N	N	N	N	N	N	N	N	N	N	N	N	N	N	N	N	N	N	N	N	N	N	N	N	N	N	N	N	N	N	N	N	N	N	N	N	N	N	N	N	N	N	N	N	N	N
Dentist guidelines for telephone assessment	N	N	N	N	N	N	N	N	N	N	N	N	N	N	N	N	N	N	N	N	N	N	N	N	N	N	N	N	N	N	N	N	N	N	N	N	N	N	N	N	N	N	N	N	N	N	N	N	N	N	N	N	N	Y	N
Medical care for refugees	N	N	N	N	N	N	N	N	N	N	N	N	N	N	N	N	N	N	N	N	N	N	N	N	N	N	N	N	N	N	N	N	N	N	N	N	N	N	Y	N	N	N	N	N	N	N	N	N	N	N	N	N	N	N	N
Alcohol ban	N	N	N	N	N	N	N	N	N	N	N	N	N	N	N	N	N	N	N	Y	N	N	N	N	N	N	N	N	N	N	N	N	N	N	N	N	N	N	N	N	N	N	N	N	N	N	N	N	N	N	N	N	N	N	N

[1] All references to Kosovo in this document should be understood to be in the context of the United Nations Security Council resolution 1244 (1999)

For peer review only - http://bmjopen.bmj.com/site/about/guidelines.xhtml

Supplementary Methods 1

For all searches, journals were searched for items published between 1 January 2020 and 17 September 2020.

1A: ProQuest Search Strategy

Coronavirus and abuse: (Covid OR Covid19 OR Coronavirus OR corona OR SARS-CoV-2 OR Covid 19) AND ("domestic violence" OR "domestic abuse" OR "intimate partner violence" OR "Gender based violence" OR "sexual violence" OR "femicide" OR “child abuse” OR “child maltreatment” OR “child neglect” or “child exploitation” OR “bullying” OR “trafficking” OR “sexual exploitation” OR “sexual abuse” OR “child marriage” or “youth violence” OR “infanticide” OR “stalking”)

1B: East View Information Service Search Strategy

((ковид OR COVID-19 OR COVID 19 OR Covid 19 OR коронавирус OR корона OR SARS-CoV-2 OR пандемия OR карантин OR изоляция) AND (домашнее насилие OR семейное насилие OR бытовое насилие OR побои OR насилие над женщинами OR сексуальное насилие OR насилие над детьми OR жестокое обращение с детьми OR эксплуатация детского труда OR издевательства OR травля OR торговля людьми OR сексуальное надругательство OR сексуальная эксплуатация OR брак с несовершеннолетними OR детский брак OR молодежное насилие OR детоубийство))

The search in Yandex.ru was based on the combinations of the following two search terms for “COVID-19” and “violence”:
(COVID-19 OR коронавирус) +(насилие)

Yandex.ru Search Strategy

((ковид | COVID-19 | COVID 19 | Covid 19 | коронавирус | корона | SARS-CoV-2 | пандемия | карантин | изоляция) +(домашнее насилие | семейное насилие | бытовое насилие | побои | насилие над женщинами | сексуальное насилие | насилие над детьми | жестокое обращение с детьми | эксплуатация детского труда | издевательства | травля | торговля людьми | сексуальное надругательство | сексуальная эксплуатация | брак с несовершеннолетними | детский брак | молодежное насилие | детоубийство))

1C: Ovid Search Strategy

(Covid OR Covid19 OR Coronavirus OR corona OR SARS-CoV-2 OR Covid 19) AND ("domestic violence" OR "domestic abuse" OR "intimate partner violence" OR "Gender based violence" OR "sexual violence" OR "femicide" OR “child abuse” OR “child neglect” or “child exploitation” OR “bullying” OR “trafficking” OR “sexual exploitation” OR “sexual abuse” OR “child marriage” or “youth violence” OR “infanticide” OR “stalking”)

For peer review only
